# Influence of Substitutions in the Binding Motif of Proline-Rich Antimicrobial Peptide ARV-1502 on 70S Ribosome Binding and Antimicrobial Activity

**DOI:** 10.3390/ijms23063150

**Published:** 2022-03-15

**Authors:** Alexandra Brakel, Andor Krizsan, Renke Itzenga, Carl N. Kraus, Laszlo Otvos, Ralf Hoffmann

**Affiliations:** 1 Faculty of Chemistry and Mineralogy, Institute of Bioanalytical Chemistry, Universität Leipzig, 04109 Leipzig, Germany; andor.krizsan@uni-leipzig.de (A.K.); ri46peva@studserv.uni-leipzig.de (R.I.); 2 Center for Biotechnology and Biomedicine, Universität Leipzig, 04109 Leipzig, Germany; 3 Aceragen Inc., Durham, NC 27709, USA; ckraus@aceragen.com (C.N.K.); laszlo@olpeconsulting.com (L.O.J.); 4 Institute of Medical Microbiology, Semmelweis University, 1085 Budapest, Hungary

**Keywords:** 70S ribosome, Chex1-Arg20, dissociation constant (K_d_), *Escherichia coli* (*E. coli*), inhibition constant (K_i_), proline-rich antimicrobial peptide (PrAMP) ARV-1502, in vitro translation, SbmA

## Abstract

Proline-rich antimicrobial peptides (PrAMPs) are promising candidates to treat bacterial infections. The designer peptide ARV-1502 exhibits strong antimicrobial effects against *Enterobacteriaceae* both in vitro and in vivo. Since the inhibitory effects of ARV-1502 reported for the 70 kDa heat-shock protein DnaK do not fully explain the antimicrobial activity of its 176 substituted analogs, we further studied their effect on the bacterial 70S ribosome of *Escherichia coli*, a known target of PrAMPs. ARV-1502 analogues, substituted in positions 3, 4, and 8 to 12 (underlined) of the binding motif D^3^KPRPYLPRP^12^ with aspartic acid, lysine, serine, phenylalanine or leucine, were tested in a competitive fluorescence polarization (FP) binding screening assay using 5(6)-carboxyfluorescein-labeled (Cf-) ARV-1502 and the 70S ribosome isolated from *E. coli* BW25113. While their effect on ribosomal protein expression was studied for green fluorescent protein (GFP) in a cell-free expression system (in vitro translation), the importance of known PrAMP transporters SbmA and MdtM was investigated using *E. coli* BW25113 and the corresponding knockout mutants. The dissociation constant (K_d_) of 201 ± 16 nmol/L obtained for Cf-ARV-1502 suggests strong binding to the *E. coli* 70S ribosome. An inhibitory binding assay indicated that the binding site overlaps with those of other PrAMPs including Onc112 and pyrrhocoricin as well as the non-peptidic antibiotics erythromycin and chloramphenicol. All these drugs and drug candidates bind to the exit-tunnel of the 70S ribosome. Substitutions of the C-terminal fragment of the binding motif YLPRP reduced binding. At the same time, inhibition of GFP expression increased with net peptide charge. Interestingly, the MIC values of wild-type and Δ*sbmA* and Δ*mdtM* knockout mutants indicated that substitutions in the ribosomal binding motif altered also the bacterial uptake, which was generally improved by incorporation of hydrophobic residues. In conclusion, most substituted ARV-1502 analogs bound weaker to the 70S ribosome than ARV-1502 underlining the importance of the YLPRP binding motif. The weaker ribosomal binding correlated well with decreased antimicrobial activity in vitro. Substituted ARV-1502 analogs with a higher level of hydrophobicity or positive net charge improved the ribosome binding, inhibition of translation, and bacterial uptake.

## 1. Introduction

Proline-rich antimicrobial peptides (PrAMPs) are promising lead compounds to overcome resistance against small-molecule antibiotics. Naturally occurring PrAMPs sequences, originally isolated primarily from insects such as fire bug (*Pyrrhocoris apterus*), milkweed bug (*Oncopeltus fasciatus*) or honey bee (*Apis mellifera*), have been optimized in recent years to improve the antibacterial activity and expand the activity spectrum while maintaining low levels of adverse effects in mammals [1,2,3]. Pharmacologically promising features of PrAMPs include the inherent stability against proteases, non-lytic mechanism of action, and a cellular uptake via bacterial transporter systems, including the SbmA transporter, allowing them to reach intracellular targets, such as chaperone DnaK and the 70S ribosome [4,5,6]. Peptide ARV-1502 (also known as Chex1-Arg20, Chex-RPDKPRPYLPRPRPPRPVR-NH_2_; Chex: 1-amino cyclohexyl carboxylic acid) and its dimer A3-APO, designed de novo starting from a sequence comparison of different insect-derived PrAMPs, are highly efficient against multidrug-resistant *Enterobacteriaceae*, especially in combination with approved antibiotics [7]. Previous studies on the intracellular target and the resulting mechanism of action focused mainly on DnaK [8,9]. ARV-1502 binds with residues YLPRP to the nucleotide binding domain of DnaK and thereby restricts the functional activity of DnaK [10]. Some ARV-1502-derived peptides showed an enhanced binding to DnaK and a better antibacterial activity against the Gram-negative bacterium *Escherichia coli* and the Gram-positive bacterium *Staphlyococcus aureus* [11]. As ARV-1502 and other PrAMPs are also active against the DnaK knockout mutant *E. coli* JW0013 ∆*dnaK*, it was suggested that further lethal bacterial targets exist [12,13]. Indeed, in addition to DnaK, PrAMPs Onc112 and Api137 inhibit the bacterial 70S ribosome in different bacteria as the main target using two alternative binding modes [6,14,15,16]. PrAMPs using the oncocin-binding mode bind medially with their N-terminus in the ribosomal exit tunnel of the 50S-subunit near the peptidyltransferase center, where it overlaps with aminoacyl-tRNA at the A site and peptidyl-tRNA at the P site of the ribosome, likely limiting the peptidyltransferase function [14,17]. In addition, the tunnel exit is blocked by the C-terminus. The oncocin-type peptides probably prevent the transition into the elongation phase [17,18]. In contrast, Api137 and apidaecin-type peptides bind to the nascent peptide exit tunnel (NPET) when the ribosome reaches the stop-codon and class one release factors (RF1 and RF2) bind to the A site [16]. Based on sequence similarities, ARV-1502 is more likely to follow the oncocin-type mechanism. Similar to DnaK binding, the N-terminal residues are crucial for binding to the ribosome, which most likely relies on hydrogen bonds and stacking interactions of aromatic and cationic residues with the nucleobases of the 23S rRNA [15,19]. Experiments using substituted oncocin analogs indicate that residues DKxxYLPRP, which are also present in ARV-1502, are important for the antimicrobial activity [12]. In general, increasingly active PrAMPs can be obtained by improving the (i) target binding, (ii) bacterial uptake rates, and (iii) protease stability [6,20,21]. In vitro screening of substituted ARV-1502 peptides showed that the antimicrobial activity against *E. coli* can be improved only by replacing the Asp^3^Lys^4^-motif mainly by hydrophobic motifs, such as Phe^3^Phe^4^, which also significantly improves the activity against *S. aureus* [11]. It needs to be mentioned that the low antibacterial activity against *S. aureus* in vitro cannot be correlated with the high efficacy of ARV-1502 against this strain in mouse models, suggesting yet additional modes of action such as immunostimulation of the mammalian host [22]. Staying with strictly antibacterial properties, here we evaluate the minimal inhibitory concentrations (MIC) of 176 singly, doubly and triply substituted ARV-1502 peptides relative to their 70S ribosome binding using a competitive fluorescence polarization assay and their ability of inhibiting in vitro protein translation.

## 2. Results

### 2.1. ARV-1502 Binds Well to E. coli 70S Ribosome

**Figure 1:**
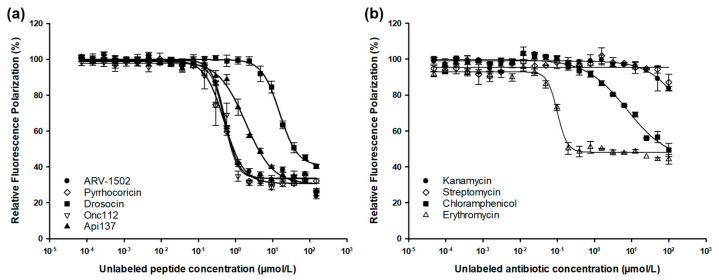
Fluorescence polarization assay using *E. coli* 70S ribosome and Cf-ARV-1502 in competition with unlabeled PrAMPs (**a**) and antibiotics (**b**). Curves were fitted to a concentration-response algorithm with a variable slope parameter [y = min + (max − min)/(1 + (x/IC50)^−Hill slope^)] by using SigmaPlot.

A recent study focusing on DnaK binding and inhibition of the ATPase activity of DnaK by ARV-1502 and its analogs could not explain the observed in vitro antibacterial activities. Thus, we broadened the scope of our research and studied the binding to the 70S ribosome isolated from *E. coli*, which was reported as one of the main intracellular targets of several PrAMPs [11]. Cf-ARV-1502 bound to the 70S ribosome preparation with a K_d_ of 201 ± 16 nmol/L (Figure 1), which was slightly higher than the K_d_ determined for *E. coli* DnaK (K_d_ = 140 ± 10 nmol/L). The inhibitory constant (K_i_) of ARV-1502 competing with Cf-ARV-1502 for the ribosomal binding site was 135 ± 10 nmol/L, which is roughly similar to the measured K_i_ values of 124 ± 2 nmol/L and 112 ± 7 nmol/L for Onc112 and pyrrhocoricin, respectively, competing with Cf-ARV-1502 (Table 1). All three peptides contain the sequence motif DKxxYLPRP supposedly important for ligand-ribosome interactions. In contrast, inhibition with drosocin (K_i_ = 3472 ± 158 nmol/L) and Api137 (K_i_ = 267 ± 31 nmol/L), which do not contain this motif, was significantly less extensive.

Antibiotics kanamycin and streptomycin, both bind to the 30S subunit, did not affect the binding of Cf-ARV-1502. However, antibiotics chloramphenicol and erythromycin known to bind to the 50S subunit competed with Cf-ARV-1502 for the same binding site, as indicated by K_i_ values of 1698 ± 481 nmol/L and 23 ± 1.6 nmol/L, respectively (Figure 1, Table 1).

### 2.2. Screening for Competitive Peptide Binders

The ribosome binding of substituted ARV-1502 analogs was tested in a fluorescence polarization (FP)-based competitive binding assay using Cf-ARV-1502. Assuming that all analogs bind only to the binding site of ARV-1502, the IC_50_ value of ARV-1502 served as a reference to identify more or less efficacious binding sequences. A ribosome concentration of 500 nmol/L, corresponding to approximately 80% of the plateau of the K_d_ curve of Cf-ARV-1502, appears to be suitable for this screening procedure. This provided as shift in the polarization (∆mP) of ~130 mP and thus a reasonable dynamic range to identify better and worse binding peptides in parallel. Since the substitutions were expected to strongly affect ribosome binding in different ways, the inhibition was recorded for a few peptides in a narrow concentration range to find the optimal peptide concentrations. IC_50_ values were mostly between 0.7 and 7 µmol/L, corresponding to K_i_ values from 0.2 to 2 µmol/L. For some peptides it was not possible to calculate an IC_50_ value, as they insufficiently inhibited ARV-1502 binding. Since the IC_50_ values varied by one order of magnitude, the screening relied on a twofold serial dilution series from 2.4 to 0.3 µmol/L. Better binding competitors should decrease the fluorescence polarization relative to ARV-1502, while less efficacious binders should show the opposite effect. Samples with minimum FP values (Cf-ARV-1502 in ribosome buffer) and maximum FP values (Cf-ARV-1502 and ribosome in ribosome buffer) were used for validation and calculation of the relative extent of fluorescence polarization. In this case, 12 minimum and 12 maximum control samples were measured in each 384-well plate corresponding to a total of 168 control samples tested. The assay quality was confirmed by Z’ factors ranging 0.86 to 0.91 and a relative standard deviation of <2%. Considering all samples on all 14 plates, the Z’ factor was 0.78 and the relative standard deviations were 1.6% and 2.7% for the maximum and minimum controls, respectively, indicating good assay quality (Supplementary material, Figure S1 and Table S1).

### 2.3. Substitutions in the Binding Motif YLPRP

**Figure 2:**
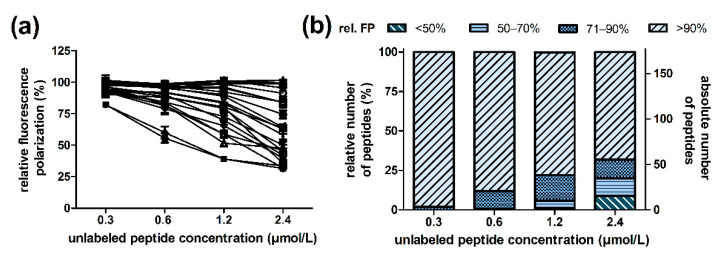
Inhibitory fluorescence polarization assay of ARV-1502 analogs using *E. coli* 70S ribosome and Cf-ARV-1502 in competition with unlabeled peptide concentrations of 0.3, 0.6, 1.2, and 2.4 µmol/L (**a**) and number of analogs able to displace Cf-ARV-1502 at the indicated concentrations (**b**). Relative FP of 25 randomly chosen peptides with different inhibitory effects (**a**). Unlabeled peptide concentration-dependent distribution of peptides for defined relative FP ranges (<50%, 50–70%, 71–90, and >90%) (**b**). Low FP values indicate stronger competition to the unlabeled ARV-1502 analog. The relative FP value of the control samples without unlabeled peptide was set to 100%.

Standard deviations obtained for the peptide samples were similar to the control samples, i.e., on average 2.3%, with the largest relative standard deviations of 9.7% observed for the highest peptide concentration. Considering the relative FP values of the highest tested peptide concentration of 2.4 µmol/L, that corresponds to 3.5-fold the IC_50_ of ARV-1502, only 23 peptides showed a strong (relative FP <60%) and 45 peptides a weak inhibitory effect (relative FP <80%) compared to a relative FP of 32.1 ± 1.3% for ARV-1502 (Table S3). About two-third of the peptides (FP values ≥90%) did not compete for the binding site of Cf-ARV-1502 (Figure 2). At a peptide concentration of 1.2 µmol/L, the relative FP values ranged for all analogs from 40.3 ± 2.2% to 108.5 ± 1.1% including ARV-1502 with a relative FP of 40.9 ± 3.9%. In this case, 21 peptides had relative FPs <80% and only three peptides showed relative FPs <60% (Figure 2, Table S3). Thus, only three analogs competed strongly with Cf-ARV-1502 for its binding site at the 70S ribosome, but none was more efficient than the parent peptide ARV-1502 itself.

**Figure 3:**
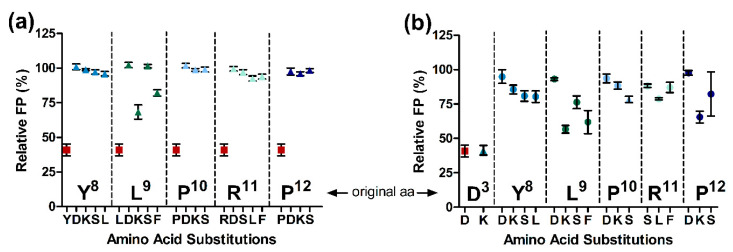
Relative FP using *E. coli* 70S ribosome and Cf-ARV-1502 competing with ARV-1502 analogs (▲) (**a**) and [D3K]-ARV-1502 analogs (●) (**b**) substituted at the indicated position of the binding motif YLPRP a concentration of 1.2 µmol/L. The relative FP values of ARV-1502 and [D3K]-ARV-1502 are indicated by ■ and ▲, respectively.

The number of substituted residues in the ARV-1502 sequence was less important for inhibition than the actual position of the substitution. Replacement of Tyr^8^ with Leu, Ser, Lys, or Asp (peptides **10** to **13**) strongly reduced the inhibition, whereas substitution of Leu^9^ with Lys (peptide **15**) or Phe (peptide **17**) were better tolerated than with Ser or Asp (Figure 3).

**Figure 4:**
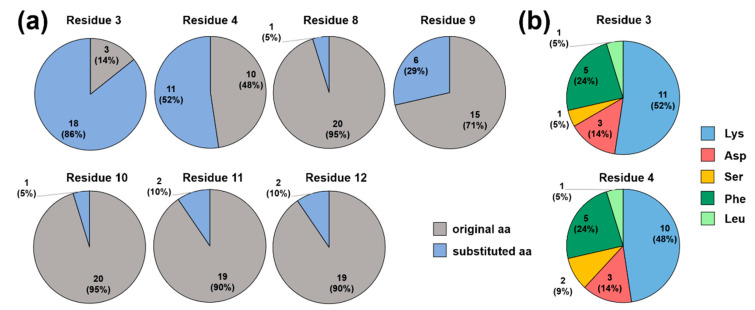
Pie diagrams indicating the substituted residues (blue) of the 21 best competing ARV-1502 analogs (relative FP < 80%; unlabeled peptide concentration of 1.2 µmol/L) for each residue (**a**) and most favorable substitutions in positions 3 and 4 (**b**).

**Figure 5:**
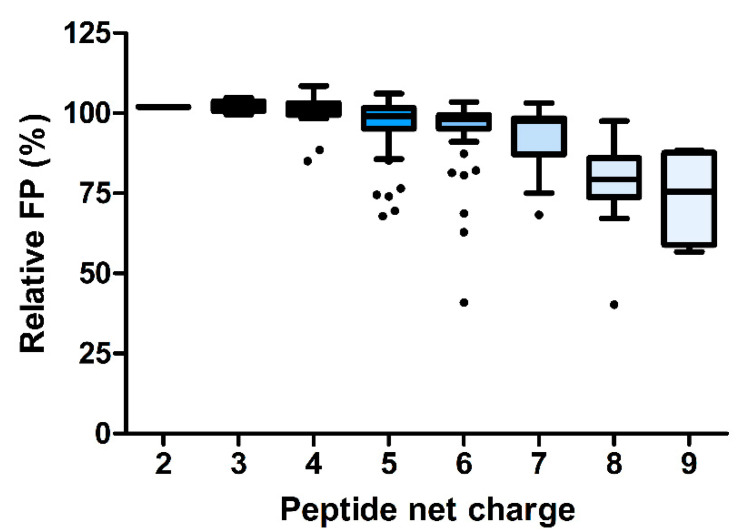
Standard boxplots (Tukey) showing the correlation of peptide net charges and relative FP values for ARV-1502 and 176 ARV-1502 analogs.

Interestingly, the inhibitory effect of ARV-1502 was not significantly reduced (relative FP <80%) when Asp^3^ or Lys^4^ were substituted with Lys and Phe (Figure 4b, blue and dark green). While Asp3Lys (peptide **2)** did not reduce the competition with Cf-ARV-1502, an additional substitution of Leu^9^ with Lys, Phe or Ser (peptides **33**, **39**, **38**), Arg^11^ with Leu (peptide **45**), or Pro^12^ with Lys (peptide **48**), weakened the binding but did not abolish it (Figure 3b). In general, peptide hydrophobicity did not correlate with the inhibitory effect, whereas a higher positive net charge slightly reduced the relative FP and thus enhanced inhibition (Figure 5).

**Figure 6:**
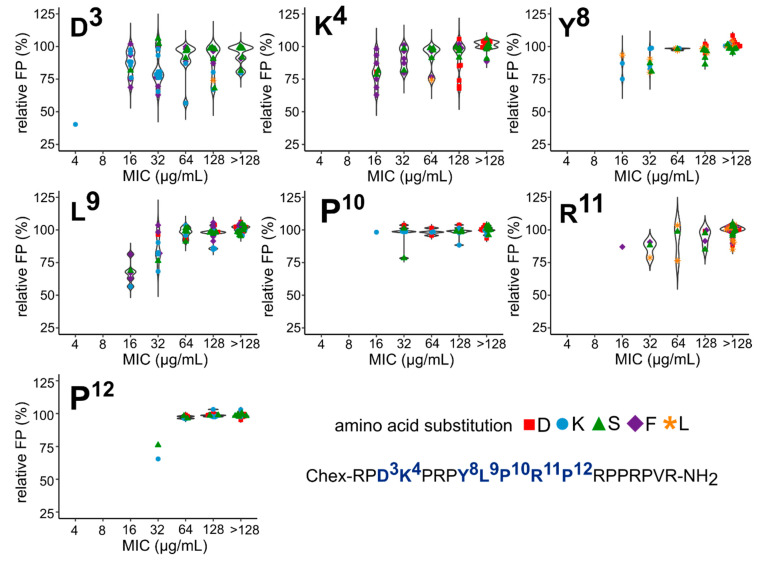
Violin plot illustrating the correlation of relative FP and MIC values for ARV-1502 analogs substituted in the binding motifs D^3^K^4^ and Y^8^LPRP^12^ with aspartic acid (■), lysine (●), serine (▲), phenylalanine (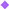) or leucine (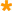).

Ribosome binding and cellular uptake are two important considerations for optimizing the antibacterial activity of peptides, but even the combination of both mechanisms does not allow predicting the MIC changes properly indicating a highly complex mode of action [23]. It is interesting to look for substitutions that change the activity and target binding in the same way. In particular, substitution of Pro^12^ typically weakened the ribosome binding and reduced the antibacterial activity at least eightfold, except for peptides **48** (Asp3Lys and Pro12Lys) and **49** (Asp3Lys and Pro12Ser) (Figure 6). However, these doubly substituted peptides were also eightfold less active than peptide **2** with an Asp3Lys substitution. Similar effects were observed for substitution of Pro^10^, which clearly reduced both binding and activity. Pro10Lys substitutions (Figure 6; blue circles) were more preferable than Pro10Asp (red squares) and Pro10Ser (green triangles) with respect to ribosome binding and MIC values. Interestingly, there were also peptides in which substitutions of ARV-1502 had the opposite effects on ribosome binding and antimicrobial activity (Supplementary material, Table S3). For example, peptides **50** (Chex-RP**SD**PRPYLPRPRPPRPVR-NH_2_) and **6** (Chex-RPD**D**PRPYLPRPRPPRPVR-NH_2_) inhibited Cf-ARV-1502 despite a low activity against *E. coli*. The opposite behavior was observed for peptides **99** (Chex-RP**FF**PRPYL**S**RPRPPRPVR-NH_2_)) and **123** (Chex-RPDKPRP**KF**PRPRPPRPVR-NH_2_) that did not inhibit the binding of Cf-ARV-1502 despite a moderate activity with MIC values of 16 to 32 µg/mL. As none of the tested peptides displaced Cf-ARV-1502 significantly better than ARV-1502, it can be concluded that the binding motif D^3^KPRPYLPRP^12^ already provides the best binding pattern considering canonical amino acids.

### 2.4. Inhibitory Effect on In Vitro Translation

**Figure 7:**
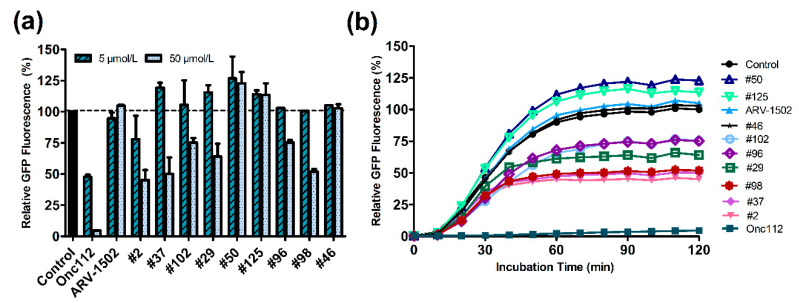
Cell-free GFP expression assay using *E. coli* 70S ribosome, sfGFP DNA template, release Factor RF1, and one PrAMP to be tested. GFP expression was monitored by the GFP fluorescence using to peptide concentrations of 5 and 50 µmol/L corresponding 2.5- and 25-fold peptide excess, respectively, (**a**) and expression kinetics for the highest peptide concentration (50 µmol/L, (**b**)).

The functional aspects of ribosome binding of ARV-1502 and its analogs on protein translation was studied by expressing GFP in a cell-free assay in the absence or presence of ARV-1502 and nine selected analogs strongly or weakly inhibiting the ribosome binding of Cf-ARV-1502 (Supplementary material, Table S5). The expression rate was monitored by the fluorescence intensity of GFP, which reached a plateau after around one hour (Figure 7). When Onc112 as control and ARV-1502 were added to the assay at a peptide concentration of 50 µmol/L corresponding to a peptide-to-ribosome ratio of 25, only Onc112 showed a strong inhibitory effect reducing GFP expression by 95% while ARV-1502 had no significant effects (Figure 7, Supplementary material, Table S5). However, peptide **2** (Asp3Lys) reduced the fluorescence intensity by 55% and still by 22% when added at a 10-fold lower concentration. Peptides **37** (binding motif: **K**KPRPY**K**PRP) and **98** (**FF**PRPYL**K**RP) reduced GFP expression by ~40% and peptide **29** (**KS**PRPYLPRP), **96** (**FF**PRPY**F**PRP), and **102** (**FF**PRPYLP**L**P) by ~25% at the higher peptide concentration (Supplementary material, Table S5). Interestingly, peptide **46** (**K**KPRPYLP**F**P) did not inhibit GFP expression despite a MIC value of 16 µg/mL.

### 2.5. Activity in ΔsbmA and ΔmdtM E. coli BW25113 Strains

PrAMPs use typically the SbmA transporter and to a lower degree also the MdtM transporter to pass the inner bacterial membrane of Gram-negative bacteria. Thus, the antimicrobial activity was also investigated for ∆*sbmA*- and *∆sbmA ∆mdtM*-knockout mutants of *E. coli* BW25113 using two different broths as culture media. *E. coli* BW25113 cultured in 25% MHB2 and 33% TSB were similarly susceptible to ARV-1502 with MIC values of 8 µg/mL and 16 µg/mL, respectively (Table 2). Generally, the substituted peptides were also less active against this *E. coli* strain when cultured in TSB, with peptides containing more basic residues being more affected by the medium. The Δ*sbmA* knockout strain was around two- to fourfold less susceptible for ARV-1502 than the wild-type strain in 25% MHB2 (MIC = 16–32 µg/mL) and 33% TSB medium (MIC = 64 µg/mL). Similar trends were observed for peptides **2**, **29**, **96, 98**, and Onc112 in 25% MHB2 medium, while the activity was more affected in 33% TSB medium. Of note, deletion of SbmA had no effect to the antibacterial activity of peptide **102**, which was already less active against the wild-type strain. The susceptibility of the double knockout strain *E. coli* BW25113 Δ*sbmA* Δ*mdtM* was further decreased for ARV-1502 again by one dilution step. The additional knockout also weakened the activity of peptide **123** and Onc112, whereas it had no effect on peptides **2**, **29**, **96**, **99**, and **102**. These results indicate that the peptides use either different transporters in spite of high sequence homologies or are active at different intracellular concentrations.

## 3. Discussion

ARV-1502 and the corresponding dimer A3-APO were designed by aligning the sequences of several insect-derived PrAMPs aiming at structures with improved antimicrobial activities. Previous studies identified residues D^3^K^4^ and Y^8^LPRP^12^ as important for DnaK binding. However, substitution of these seven residues with basic (Lys), acidic (Asp), hydrophilic (Ser), aliphatic (Leu), and aromatic canonical amino acids (Phe) showed only minor effects on DnaK binding [11]. The ATPase activity of DnaK and the chaperone activity of DnaK in a refolding assay using DnaK and co-chaperones were affected, but these data do not correlate to the MIC values obtained for *E. coli* and *S. aureus*. Thus, this report focuses on ribosome binding, as the YLPRP-motif is crucial for both ribosome binding, as previously shown for PrAMPs Onc112 and pyrrhocoricin [24], and bacterial uptake.

Indeed, the dissociation constants of Cf-labeled ARV-1502, pyrrhocoricin, and Onc112 to the 70S ribosome of *E. coli* were very similar and the K_i_ values among these closely related PrAMPs indicated a similar binding side, as anticipated from the binding motif.

In general, ARV-1502 strongly displaced chloramphenicol and erythromycin indicating a dual mechanism in inhibiting translation. The macrolide antibiotic erythromycin primarily binds the upper part of the ribosomal exit tunnel inhibiting translocation of the elongation factor EF-G during translation and thus displays a mechanism resembling that of ARV-1502. In addition, similar to chloramphenicol that binds directly to the A-site within the PTC, ARV-1502 blocks the attachment site of the aminoacyl residue of the A-site tRNA [25]. Similar results were observed for Onc112, also containing the YLPRP binding motif [14]. In spite of overlapping binding sites, a checkerboard assay with ARV-1502 and chloramphenicol did not indicate synergistic or antagonistic effects (Supplementary material, Table S6). Slightly higher FICIs were observed for peptide 2, which may show that peptide 2 and chloramphenicol compete in cells for the same binding site. Similarly, kanamycin, an antibiotic that targets the 30S subunit and the non-ribosome-acting antibiotic ciprofloxacin inhibiting gyrase and DNA replication showed neither synergistic nor antagonistic effects. Thus, it can be assumed that the binding sites and modes of action are different.

Binding of PrAMPs deep in the ribosomal exit tunnel with reversed orientation to the nascent polypeptide chain is mediated mainly via hydrophobic interactions, stacking interactions, and hydrogen bonds with the nucleic bases and 23S rRNA [26]. Replacing amino acids mainly involved in these interactions with amino acids of different physicochemical properties can allow either strengthen or weaken these essential interactions. The ARV-1502 library underlined the importance of the YLPRP motif for ribosomal binding, as none of the substitutions improved the binding. Substitutions of Leu^9^ were better tolerated than that of other residues. For example, Leu9Lys and Leu9Phe were weak competitors in contrast to substitutions at Tyr^8^, Pro^10^, Arg^11^, and Pro^12^. The hydrophobic aromatic character of Phe allows the formation of both π-stacking interactions and hydrophobic interactions, while Lys can form salt bridges with the oppositely charged phosphate groups of the rRNA. Thus, basic residues appear to be preferable than acidic residues. Although Asp^3^ and Lys^4^ residues located near the N-terminus entered most deeply into the exit tunnel, they seem to be less important for ribosome binding. Even the extreme Asp3Lys substitution in peptide **2** (**K**KPRPYLPRP) did not alter the K_i_ value. As substitutions with Phe, Leu, and Ser were also well-tolerated, this position might be well-suited to further improve peptide properties without interfering with target binding. The increasing net charge of peptide **2** (+8 compared to +6 for ARV-1502) improved both the antimicrobial activity against *Escherichia coli* and the inhibitory effect on in vitro translation (Table 3).

Surprisingly, despite similar K_i_ values and similar binding sites of ARV-1502 and Onc112, most likely driven by the binding motif YLPRP, Onc112 fully inhibited GFP expression, but ARV-1502 not at all. This confirms previous reports on ARV-1502 allowing in vitro translation under different experimental conditions, while its dimerized version A3-APO was able to inhibit GFP expression with an IC_50_ of 1.6 µmol/L. This value is still ~10-fold higher than the IC_50_ of Onc112 (IC_50_ = 0.15 µmol/L) (Supplementary material, Figure S2) [4]. The difference between monomer and dimer could be related to the doubled net charge, as similar effects occurred with ARV-1502 analogs, or the increased size allowing further interactions in the exit-tunnel. Interestingly, A3-APO appears to be a strong competitor of Cf-Onc112, Cf-ARV-1502, and Cf-Api137 with K_i_ values of 24 nmol/L, 55 nmol/L, and 120 nmol/L, respectively, compared to the K_i_ values of 120 nmol/L, 73 nmol/L, and 3140 nmol/L, respectively, obtained for ARV-1502. The monomer was a very weak competitor of Cf-Api137 (Supplementary material, Figure S3). It needs to be mentioned that the dimer exhibits a broader activity spectrum than the monomer, but the latter shows lower MIC values against sensitive strains and is active at lower doses in vivo [27].

Higher charged peptides with good ribosome binding improved the inhibitory effect compared to ARV-1502 but were still less significant than Onc112 (Figure 7b). The effect of polybasic sequences slowing down and restricting the translation mechanism by interactions of the positively charged peptide with the negative electrostatic potential of the exit tunnel was previously reported [28,29,30,31]. Strong electrostatic interactions cancel translation before the elongation step. However, an increase of the net charge is not the only criterion, as the Leu9Lys substitution in peptide **125** (DKPRP**SK**PRP) slightly improved GFP translation. Conversely, peptide **98** (**FF**PRPYL**K**RP) that was a poor competitor of Cf-ARV-1502 in the ribosomal binding, suppressed GFP expression by around 50% suggesting another possible binding site that could be confirmed prospectively by experiments using additional Cf-labeled peptides.

The effect on in vitro translation did not always correlate with antimicrobial activity against *E. coli*. Despite lower inhibitory effects and weaker ribosome binding, peptides ARV-1502, **2**, and 98 were equally active when *E. coli* was cultured in 25% MHB2 or 33% TSB indicating a more favorable transporter-mediated uptake. The MIC values of all peptides were higher in 33% TSB than in 25% MHB2 with peptides containing a higher number of Lys residues being slightly more affected. This might be related to the higher chloride and phosphate concentrations in TSB disturbing the interaction of the peptides with the negatively charged bacterial surface. Previous studies already demonstrated lower uptake rates in TSB compared to MHB cultivation conditions [20]. Previous studies identified the SbmA transporter as most relevant for the uptake of PrAMPs, while oncocin-like PrAMPs, such as Onc112 and ARV-1502, use additionally the MdtM transporter system [32,33,34]. With the exception of peptide **102**, *E. coli* BW25113 Δ*sbmA* was less susceptible to all peptides when cultivated in 25% MHB2 and even more pronounced in 33% TSB, as also observed for other PrAMPs including pyrrhocoricin and drosocin [20]. The double knockout mutant BW25113 Δ*sbmA* Δ*mdtM* reduced the activity of ARV-1502 and peptide **123** further. A striking feature of peptides depending less on a SbmA-mediated uptake, was the increased hydrophobicity due to the insertion of Phe and the absence of positively charged residues Lys^3^ or Arg^10^. Since MdtM is a Na^+^/K^+^:H^+^ antiporter and efflux pump driven by an electrochemical gradient, basic residues might have a crucial influence on this transport process [35,36]. Particularly remarkable was the activity of peptide **102**, which was similar for the wild-type and knockout strains. Interestingly, a similar observation was reported for A3-APO with a MIC of 32 µg/mL in 33% TSB [34]. These similar activities independent of the known transporters, could indicate that these peptides enter the cytoplasm either by passive diffusion or rely on other transporter active mechanisms. By replacing only a few amino acids with Phe or Leu and simultaneously preserving the PRP-motif in the peptide sequence, it was possible to increase the hydrophobicity without destroying the amphipathic character. Such substitutions may allow peptides to enter bacterial cells at least partially by passive diffusion and thus depend less on transporter-mediated uptake. An increased hydrophobicity while retaining the positive charge can improve permeabilization due to strong LPS interactions or trigger destructive membrane effects improving the cellular uptake [37]. Indeed, the increasingly basic A3-APO dimer has a stronger effect on the *E. coli* membrane than the monomer ARV-1502 due to stronger electrostatic interactions [38].

## 4. Materials and Methods

### 4.1. Materials

Reagents were obtained from the following manufacturers: AppliChem GmbH (Darmstadt, Germany): Dithiothreitol (DTT, ≥99%) and HEPES (>99.5%); Carl Roth GmbH & Co. KG (Karlsruhe, Germany): Chloramphenicol (98.8%), kanamycin sulfate (>750 I.U/mg), Lysogeny broth (LB), lysozyme (≥45,000 FIP U/mg), putrescine (≥99%), spermidine (≥99%), and zirconia/silica beads (0.1 mm dia); Honeywell FlukaTM (Seelze, Germany): Calcium chloride (≥99.5%), ciprofloxacin (98.0%) and magnesium chloride hexahydrate (>99%); SERVA electrophoresis GmbH (Heidelberg, Germany): Tween^®^ 20 (pure); Sigma Aldrich Chemie GmbH (Taufkirchen, Germany): ammonium chloride (≥99.5%), 5(6)-carboxyfluorescein (for fluorescence), caseine (from bovine milk), disodium hydrogen phosphate (≥99%), magnesium acetate tetrahydrate (≥99%), 2-mercaptoethanol (≥99%), potassium hydroxide (≥90%), potasssium phosphate (≥99%), and sodium chloride (≥99.5%); Thermo Fisher Scientific Inc. (Darmstadt, Germany): DNase I (RNase-free, 1 U/μL) and potassium glutamate (≥97%).

Water (resistance R > 18 mΩ/cm; total organic content <10 ppb) was purified by a PureLab Ultra Analytic system (ELGA Lab Water, Celle, Germany).

Peptides: ARV-1502 acetate was obtained from PolyPeptide Laboratories (SanDiego, CA, USA) as white powder with a purity of 97.3% according to RP-HPLC. The residual TFA content was 0.05%. The identity was further confirmed by amino acid analysis (Asx, Pro, Val, Leu, Tyr, Lys, and Arg). The 182 substituted analogs of ARV-1502 were obtained from ABclonal, Inc. (Woburn, MA, USA). These peptides were purified by RP-HPLC using an acetonitrile gradient in the presence of 0.1% TFA. Masses were confirmed by ESI-MS and the purities (>80%) were determined by RP-HPLC recording the absorbance at 214 nm. Peptides containing a N-terminal 5(6)-carboxyfluorescein-label were synthesized in-house by Fmoc/^t^Bu-chemistry on Rink amide resin and purified by RP-HPLC using an acetonitrile gradient in the presence of 0.1% TFA. Masses were confirmed by ESI-MS and the purities (>95%) were determined by RP-HPLC recording the absorbance at 214 nm.

### 4.2. Preparation of E. coli 70S Ribosomes

*E. coli* 70S ribosomes were prepared using a previously described protocol that was slightly modified [4,6]. Briefly, *E. coli* BW25113 was cultivated in Luria-Bertani (LB) medium and cells were harvested after reaching an optical density of ~4 at 600 nm by centrifugation (5000× *g*, 15 min, 4 °C, Rotor JLA 8.100, Avanti J-20 XP, Beckmann Coulter, Krefeld, Germany). The cell pellets were frozen and stored at −80 °C. Cells were resuspended in ribosome buffer (2 mL/g cells; 20 mmol/L HEPES-KOH, 6 mmol/L MgCl_2_, 30 mmol/L NH_4_Cl, 4 mmol/L 2-mercaptoethanol, pH 7.6). Lysozyme (0.25 g/L cell suspension) was added, and the mixture was incubated on ice for 30 min. Cells were disrupted using the bead mill homogenizer FastPrep-24™ 5G (40 s, 4 m/s, 6 cycles, MP Biomedicals Germany GmbH, Eschwege, Germany) and zirconia/silica beads (0.1 mm diameter). The lysate was centrifuged (1500× *g*, 5 min, 4 °C, Rotor S4180, Allegra 21R, Beckmann Coulter) and the supernatant was incubated with DNase (5 U/mL) on ice for 60 min. The cell debris was removed by two centrifugation steps (16,000× *g*, 30 min, 4 °C followed by 32,000× *g*, 60 min, 4 °C, Rotor JA 30.50 Ti, Avanti J-30I, Beckmann Coulter). The ribosome was pelleted by ultracentrifugation (165,000× *g*, 17 h, 4 °C, Rotor 70 Ti, Optima LE-80K, Beckmann Coulter), resuspended in ribosome buffer (0.1 mL/g pellet), and stored at −80 °C. The ribosome concentration was determined by recording the absorbance of RNA at 260 nm (NanoPhotometer NP80, Implen GmbH, München, Germany) assuming that 1 AU corresponds to a ribosome concentration of 28 nmol/L. The molecular weight of the *E. coli* 70S ribosome was assumed to be 2.3 MDa.

### 4.3. Determination of Dissociation and Inhibitory Constants

Dissociation and inhibitory constants were determined in black 384-well-plates (Greiner Bio-One GmbH, Frickenhausen, Germany) blocked with 0.5% (*w/v*) casein in phosphate buffered saline (PBS, 8.8 mmol/L Na_2_HPO_4_×12 H_2_O, 1.2 mmol/L KH_2_PO_4_, 0.3 mol/L NaCl, pH 7.4) containing 0.05% (*w/v*) Tween^®^ 20 (PBST) overnight at 4 °C and washed three times with PBST.

Dissociation constants (K_d_) were determined using a twofold serial dilution series in 23 steps from 30 µmol/L to 7 pmol/L of ribosome in ribosome buffer (20 µL/well) and 5(6)-carboxyfluorescein-labeled peptide was added (20 µL; final concentration 20 nmol/L). After centrifugation (2 min, 500× *g*, Rotor S2096, Allegra^TM^ 21R, Beckmann Coulter), the plate was incubated at 28 °C in the dark for 90 min. The extent of fluorescence polarization was recorded using an excitation wavelength (λ_ex_) of 485 nm and an emission wavelength (λ_em_) of 535 nm on a PARADIGM^TM^ microplate reader (Beckmann Coulter).

Inhibitory constants (K_i_) were determined using a twofold serial dilution series of the unlabeled peptide from 150 µmol/L to 70 pmol/L in ribosome buffer (20 µL). Ribosome solution was added (10 µmol/L) and the plate was incubated after centrifugation (2 min, 500× *g*, Rotor S2096, Allegra^TM^ 21R, Beckmann Coulter) at 28 °C for 90 min. Cf-labeled peptide was added (10 µL; final concentration of 20 nmol/L; final ribosome concentration of 0.5 µmol/L) and the plate was centrifuged and incubated again (90 min, 28 °C, dark). Fluorescence polarization was recorded (λ_ex_ = 485 nm, λ_em_ = 535 nm) on a PARADIGM^TM^ microplate reader. K_d_ and IC_50_ values were calculated by fitting the data with a variable slope parameter [y = min + (max − min)/(1 + (x/Kd) ^−Hill slope^) ] using SigmaPlot 13 (Systat Software Inc., San Jose, CA, USA). The obtained IC_50_ values were used to calculate the K_i_ values [39].

### 4.4. Screening for Competitive Binder Peptides

*E. coli* 70S ribosomes and Cf-ARV-1502 were incubated with four different concentrations of unlabeled peptides chosen based on the IC_50_ curves of reference peptide ARV-1502. Each peptide was diluted in ribosome buffer to obtain a concentration of 4.8 µmol/L and transferred to a 384-well-plate. Peptides were then twofold serially diluted in four steps from 4.8 to 0.6 µmol/L (20 µL). The ribosome extract was diluted in ribosome buffer to reach a concentration of 2 µmol/L and 10 µL were added to each well to obtain a final ribosome concentration of 0.5 µmol/L. Plates were centrifuged (2 min, 500× *g*, Rotor S2096, Allegra^TM^ 21R, Beckmann Coulter) and incubated at 28 °C for 90 min. Cf-labeled ARV-1502 was added (10 µL; final concentration of 20 nmol/L) and the plates were again centrifuged and incubated (28 °C, 90 min). After incubation, fluorescence polarization was recorded on a PARADIGM^TM^ microplate reader (λ_ex_ = 485 nm, λ_em_ = 535 nm).

On each plate 12 minimum (30 µL ribosome buffer and 10 µL Cf-ARV-1502) and 12 maximum (20 µL ribosome buffer, 10 µL ribosome solution and 10 µL Cf-ARV-1502) control samples were added. Fluorescence polarization of screening samples was normalized to the fluorescence polarization of these control samples. All screening samples were measured in triplicates and the whole experiment was repeated once on another day.

### 4.5. Antimicrobial Activity

MIC values were determined using a liquid broth micro dilution assay in sterile 96-well plates (polystyrene F-bottom, Greiner Bio-One GmbH) and a total volume of 100 μL per well. Aqueous peptide solutions (10 g/L) were serially twofold diluted in 25% Mueller-Hinton broth 2 (25% MBH2) or 33% tryptic soy broth (33% TSB) starting at a peptide concentration of 128 mg/L (50 μL/well). Overnight cultures of bacteria grown in 25% MHB2 (or 33% TSB) were diluted 30-fold in 25% MHB2 (or 33% TSB). After an incubation period of 4 h (37 °C, 200 rpm), cells were diluted to 1.5 × 10^7^ cfu/mL, based on a McFarland test, and 50 μL were added to each well (final concentration of 7.5 × 10^6^ cfu per well). The plates were incubated (37 °C, 20 h) and the optical density was recorded at 595 nm using a microplate reader (PARADIGM^TM^, Beckmann Coulter). The MIC was defined as the lowest peptide concentration preventing visible bacterial growth.

### 4.6. Cell-Free Protein Expression Assay

Possible effects of ARV-1502 and its substituted analogs on the in vitro translation of the green fluorescent protein (GFP) were probed using the NEB PureExpress Delta RF123 Kit (New England Biolabs, Ipswich, MA, USA). The sfGFP DNA template was amplified from pY71sfGFP plasmid by PCR introducing an UAA stop codon. Release factor 1 (RF1) was 50fold diluted in Pure System Buffer (PSB) containing magnesium acetate (1 mol/L), monopotassium phosphate (0.5 mol/L, pH 7.3), potassium glutamate (1 mol/L), NH_4_Cl (1 mol/L), calcium chloride (0.5 mol/L), spermidine (1 mol/L), putrescine (0.1 mol/L), and DTT (0.1 mol/L). Each reaction used 35 ng of sfGFP template. Peptides were added at a final concentration of 5 µmol/L and 50 µmol/L. The reaction was started by adding Kit solutions A (2 µL) and B (1.5 µL), diluted RF1 (0.5 µL), sfGFP template (0.25 µL or water as negative control), PSB (0.25 µL), and peptide (0.5 µL or water), mixed, transferred into a black 384-well plate (flat bottom, Greiner Bio-One GmbH), and incubated (37 °C, 2 h). The fluorescence was recorded every 10 min (λ_exc_ = 485 nm, λ_em_ = 535 nm) in a microplate reader (Gemini EM, Molecular Devices LLC, San Jose, CA, USA).

### 4.7. Checkerboard Assay

Synergy was tested in sterile 96-well plates (polystyrene F-bottom, Greiner Bio-One GmbH) using a total volume of 100 μL per well. Peptides were added in a twofold dilution series starting with 2 × MIC. Chloramphenicol, kanamycin or ciprofloxacin were added orthogonal in a twofold dilution series starting with 2 × MIC. Overnight cultures of *E. coli* BW25113 grown in 25% MHB2 were diluted 30-fold and incubated (37 °C, 200 rpm, 4 h). Cells were diluted to 1.5 × 10^7^ cfu/mL and 50 μL were added to each well (final concentration of 7.5 × 10^6^ cfu per well). Plates were incubated (37 °C, 20 h) and the optical density recorded at 595 nm using a microplate reader (Victor3, Perkin Elmer Inc., Waltham, MA, USA). The fractional inhibitory concentration index (FICI) was calculated by the equation FICI = FIC_A_ + FIC_B_ = (A/MIC_A_) + (B/MIC_B_), where MIC_A/B_ are the MICs of an antimicrobial peptide (A) and one of the three tested antibiotics (B) alone and A and B are the MICs of combined antimicrobials. Synergy was defined as FICI ≤ 0.5, antagonism as FICI ≥ 4 and additive or indifference as FICI between 0.5 and 4.

## 5. Conclusions

This study suggests that inhibition of the 70S ribosome by ARV-1502 does not follow the oncocin nor the apidaecin-type mechanism on transcription inhibition but may rather rely on a third type of mechanism in spite of sharing a high sequence homology and an identical binding motif with Onc112. Even a single substitution with a non-homologous canonical amino acid in the binding motif abolished the binding to the ARV-1502 binding site on the *E. coli* 70S ribosome. A potential alternative binding site cannot be ruled out and should be investigated in further studies. Despite the poor competition at the ARV-1502 and Onc112 binding sites, some analogs were able to inhibit in vitro translation more efficiently than ARV-1502 or acted independent of the known transporters SbmA and MdtM. Lys^3^ and Asp^4^ residues were identified as possible exchange positions where optimization can be performed without disrupting the target action.

## Figures and Tables

**Table 1:** Inhibition constants (K_i_) determined for *E. coli* 70S ribosome and Cf-labeled ARV-1502 in competition with different PrAMPs and antibiotics.

Peptide.	Sequence	K_i_ (nmol/L)
ARV-1502	ChexRPDKPRPYLPRPRPPRPVR-NH_2_	135 ± 10
Pyrrhocoricin	VDK GSYLPRPTPPRPIYNRN	112 ± 7
Drosocin	GKPRPYSPRPTSHPRPIRV	3472 ± 158
Onc112	VDKPP YLPRPRPPRrIYNr-NH_2_	124 ± 2
Api137	gu-ONNRPVYIPRPRPPHPRL-OH	267 ± 31
**Antibiotic**	**Ribosome Binding Site**	**K_i_ (nmol/L)**
Kanamycin	30S subunit	no fit
Streptomycin	30S subunit	no fit
Chloramphenicol	50S subunit	1698 ± 481
Erythromycin	50S subunit	23 ± 1.6

Chex- 1-amino cyclohexyl carboxylic acid, gu–1,1,3,3 tetramethyl guanidine, O-l-ornithine, r-d-arginine.

**Table 2:** MIC values determined for *E. coli* BW25113, BW25113 ∆*sbmA*, and BW25113 ∆*sbmA* ∆*mdtM*. Bacteria were cultured in 25% MHB2 or 33% TSB. Peptides were tested at least as duplicates on two different days, except for peptides marked with an asterisk (*), that were measured once on two days or with two asterisks (**), that were measured in duplication only on one day.

Peptide	Sequence Motif	Minimal Inhibitory Concentration (µg/mL)					
		25% MHB2			33% TSB		
		*wt*	∆*sbmA*	∆*sbmA* ∆*mdtM*	*wt*	∆*sbmA*	∆*sbmA* ∆*mdtM*
ARV-1502	DK YLPRP	8	16–32	64	16	64	128
**#2**	**K**K YLPRP	4	8	16	32–64	64	64
**#29**	**KS** YLPRP	16	32	32	32	128	128
**#37**	**K**K Y**K**PRP	16	16 **	32 **	64	≥128	>128
**#46**	**K**K YLP**F**P	16	32 **	32 **	64 *	≥128 *	>128
**#50**	**SD** YLPRP	128	>128	>128	64	>128	>128
**#96**	**FF** Y**F**PRP	16	16–32	32 **	32 *	≥128	≥128
**#98**	**FF** YL**K**RP	8–16	32	32	32	128	128
**#99**	**FF** YL**S**RP	16–32	32	32	64	128	128
**#102**	**FF** YLP**L**P	32–64	64	64	64	64	64–128
**#123**	DK **KF**PRP	32	64	128	128	>128	>128
**#125**	DK **SK**PRP	128	>128	>128	>128	>128	>128
Onc112	DK YLPRP	8	16–32	64	16	64	128

**Table 3:** Comparison of competitive ribosome binding against Cf-ARV-1502, MIC values obtained for *E. coli* BW25113 in 25% MHB2, and inhibition of the in vitro translation of GFP for ARV-1502 analogs with interesting properties. The strength of the different effects is indicated by the color code, i.e., red, orange, yellow, and green indicating no, moderate, strong and very strong effects, respectively.

Peptide.	Sequence Motif	Ribosome Binding (%)	MIC (µg/mL)	In Vitro Translation (%)	Charge State
**ARV-1502**	DK YLPRP	40.9 ± 3.9	8	105.8 ± 0.9	+6
**#2**	**K**K YLPRP	40.3 ± 2.2	4	53.2 ± 8.2	+8
**#29**	**KS** YLPRP	78.5 ± 3.3	16	74.3 ± 10.1	+7
**#37**	**K**K Y**K**PRP	56.7 ± 2.6	16	63.4 ± 13.3	+9
**#46**	**K**K YLP**F**P	87.1 ± 3.4	16–32	96.6 ± 3.1	+7
**#50**	**SD** YLPRP	67.9 ± 4.8	128	113.7 ± 9.1	+5
**#96**	**FF** Y**F**PRP	62.9 ± 4.8	16	77.2 ± 1.9	+6
**#98**	**FF** YL**K**RP	98.3 ± 1.5	8–16	56.2 ± 2.1	+7
**#102**	**FF** YLP**L**P	76.5 ± 5.6	32–64	78.9 ± 3.5	+5
**#125**	DK **SK**PRP	86.3 ± 2.5	128	104.3 ± 9.3	+7
**Onc112**	DK YLPRP	32.1 ± 1.0	8	4.9 ± 0.3	+5

## Data Availability

Not applicable.

## Supplementary Materials

The following supporting information can be downloaded at: https://www.mdpi.com/article/10.3390/ijms23063150/s1.

## References

[1] Scocchi M., Tossi A., Gennaro R. (2011). Proline-rich antimicrobial peptides: Converging to a non-lytic mechanism of action. Cell. Mol. Life Sci..

[2] Li W., Tailhades J., O’Brien-Simpson N.M., Separovic F., Otvos L., Hossain M.A., Wade J.D. (2014). Proline-rich antimicrobial peptides: Potential therapeutics against antibiotic-resistant bacteria. Amino Acids.

[3] Otvos L. (2002). The short proline-rich antibacterial peptide family. Cell. Mol. Life Sci..

[4] Krizsan A., Volke D., Weinert S., Sträter N., Knappe D., Hoffmann R. (2014). Insect-derived proline-rich antimicrobial peptides kill bacteria by inhibiting bacterial protein translation at the 70S ribosome. Angew. Chem..

[5] Runti G., Lopez Ruiz M.D.C., Stoilova T., Hussain R., Jennions M., Choudhury H.G., Benincasa M., Gennaro R., Beis K., Scocchi M. (2013). Functional characterization of SbmA, a bacterial inner membrane transporter required for importing the antimicrobial peptide Bac7(1-35). J. Bacteriol..

[6] Kolano L., Knappe D., Volke D., Sträter N., Hoffmann R. (2020). Ribosomal Target-Binding Sites of Antimicrobial Peptides Api137 and Onc112 Are Conserved among Pathogens Indicating New Lead Structures to Develop Novel Broad-Spectrum Antibiotics. Chembiochem.

[7] Otvos L., de Olivier Inacio V., Wade J.D., Cudic P. (2006). Prior antibacterial peptide-mediated inhibition of protein folding in bacteria mutes resistance enzymes. Antimicrob. Agents Chemother..

[8] Otvos L., Rogers M.E., Consolvo P.J., Condie B.A., Lovas S., Bulet P., Blaszczyk-Thurin M. (2000). Interaction between Heat Shock Proteins and Antimicrobial Peptides. Biochemistry.

[9] Knappe D., Goldbach T., Hatfield M.P.D., Palermo N.Y., Weinert S., Sträter N., Hoffmann R., Lovas S. (2016). Proline-rich Antimicrobial Peptides Optimized for Binding to Escherichia coli Chaperone DnaK. Protein Pept. Lett..

[10] Zahn M., Berthold N., Kieslich B., Knappe D., Hoffmann R., Sträter N. (2013). Structural studies on the forward and reverse binding modes of peptides to the chaperone *DnaK*. J. Mol. Biol..

[11] Brakel A., Kolano L., Kraus C.N., Otvos L., Hoffmann R. (2022). Functional Effects of ARV-1502 Analogs Against Bacterial Hsp70 and Implications for Antimicrobial Activity. Front. Chem..

[12] Knappe D., Ruden S., Langanke S., Tikkoo T., Ritzer J., Mikut R., Martin L.L., Hoffmann R., Hilpert K. (2016). Optimization of oncocin for antibacterial activity using a SPOT synthesis approach: Extending the pathogen spectrum to Staphylococcus aureus. Amino Acids.

[13] Czihal P., Knappe D., Fritsche S., Zahn M., Berthold N., Piantavigna S., Müller U., van Dorpe S., Herth N., Binas A. (2012). Api88 is a novel antibacterial designer peptide to treat systemic infections with multidrug-resistant Gram-negative pathogens. ACS Chem. Biol..

[14] Krizsan A., Prahl C., Goldbach T., Knappe D., Hoffmann R. (2015). Short Proline-Rich Antimicrobial Peptides Inhibit Either the Bacterial 70S Ribosome or the Assembly of its Large 50S Subunit. Chembiochem.

[15] Seefeldt A.C., Graf M., Pérébaskine N., Nguyen F., Arenz S., Mardirossian M., Scocchi M., Wilson D.N., Innis C.A. (2016). Structure of the mammalian antimicrobial peptide Bac7(1-16) bound within the exit tunnel of a bacterial ribosome. Nucleic Acids Res..

[16] Florin T., Maracci C., Graf M., Karki P., Klepacki D., Berninghausen O., Beckmann R., Vázquez-Laslop N., Wilson D.N., Rodnina M.V. (2017). An antimicrobial peptide that inhibits translation by trapping release factors on the ribosome. Nat. Struct. Mol. Biol..

[17] Roy R.N., Lomakin I.B., Gagnon M.G., Steitz T.A. (2015). The mechanism of inhibition of protein synthesis by the proline-rich peptide oncocin. Nat. Struct. Mol. Biol..

[18] Gagnon M.G., Roy R.N., Lomakin I.B., Florin T., Mankin A.S., Steitz T.A. (2016). Structures of proline-rich peptides bound to the ribosome reveal a common mechanism of protein synthesis inhibition. Nucleic Acids Res..

[19] Graf M., Mardirossian M., Nguyen F., Seefeldt A.C., Guichard G., Scocchi M., Innis C.A., Wilson D.N. (2017). Proline-rich antimicrobial peptides targeting protein synthesis. Nat. Prod. Rep..

[20] Holfeld L., Hoffmann R., Knappe D. (2017). Correlating uptake and activity of proline-rich antimicrobial peptides in Escherichia coli. Anal. Bioanal. Chem..

[21] Noto P.B., Abbadessa G., Cassone M., Mateo G.D., Agelan A., Wade J.D., Szabo D., Kocsis B., Nagy K., Rozgonyi F. (2008). Alternative stabilities of a proline-rich antibacterial peptide in vitro and in vivo. Protein Sci..

[22] Ostorhazi E., Holub M.C., Rozgonyi F., Harmos F., Cassone M., Wade J.D., Otvos L. (2011). Broad-spectrum antimicrobial efficacy of peptide A3-APO in mouse models of multidrug-resistant wound and lung infections cannot be explained by in vitro activity against the pathogens involved. Int. J. Antimicrob. Agents.

[23] Kolano L., Knappe D., Berg A., Berg T., Hoffmann R. (2021). Effect of Amino Acid Substitutions on 70S Ribosomal Binding, Cellular Uptake, and Antimicrobial Activity of Oncocin Onc112. Chembiochem.

[24] Graf M., Wilson D.N. (2019). Intracellular Antimicrobial Peptides Targeting the Protein Synthesis Machinery. Adv. Exp. Med. Biol..

[25] Svetlov M.S., Plessa E., Chen C.-W., Bougas A., Krokidis M.G., Dinos G.P., Polikanov Y.S. (2019). High-resolution crystal structures of ribosome-bound chloramphenicol and erythromycin provide the ultimate basis for their competition. RNA.

[26] Seefeldt A.C., Nguyen F., Antunes S., Pérébaskine N., Graf M., Arenz S., Inampudi K.K., Douat C., Guichard G., Wilson D.N. (2015). The proline-rich antimicrobial peptide Onc112 inhibits translation by blocking and destabilizing the initiation complex. Nat. Struct. Mol. Biol..

[27] Ostorhazi E., Voros E., Nemes-Nikodem E., Pinter D., Sillo P., Mayer B., Wade J.D., Otvos L. (2013). Rapid systemic and local treatments with the antibacterial peptide dimer A3-APO and its monomeric metabolite eliminate bacteria and reduce inflammation in intradermal lesions infected with Propionibacterium acnes and meticillin-resistant Staphylococcus aureus. Int. J. Antimicrob. Agents.

[28] Lu J., Kobertz W.R., Deutsch C. (2007). Mapping the electrostatic potential within the ribosomal exit tunnel. J. Mol. Biol..

[29] Charneski C.A., Hurst L.D. (2013). Positively charged residues are the major determinants of ribosomal velocity. PLoS Biol..

[30] Wang J., Karki C., Xiao Y., Li L. (2020). Electrostatics of Prokaryotic Ribosome and Its Biological Implication. Biophys. J..

[31] Requião R.D., de Souza H.J.A., Domitrovic T., Palhano F.L. (2016). Increased ribosome density associated to positively charged residues is evident in ribosome profiling experiments performed in the absence of translation inhibitors. RNA Biol..

[32] Paulsen V.S., Mardirossian M., Blencke H.-M., Benincasa M., Runti G., Nepa M., Haug T., Stensvåg K., Scocchi M. (2016). Inner membrane proteins YgdD and SbmA are required for the complete susceptibility of Escherichia coli to the proline-rich antimicrobial peptide arasin 1(1-25). Microbiology.

[33] Mattiuzzo M., Bandiera A., Gennaro R., Benincasa M., Pacor S., Antcheva N., Scocchi M. (2007). Role of the Escherichia coli SbmA in the antimicrobial activity of proline-rich peptides. Mol. Microbiol..

[34] Krizsan A., Knappe D., Hoffmann R. (2015). Influence of the yjiL-mdtM Gene Cluster on the Antibacterial Activity of Proline-Rich Antimicrobial Peptides Overcoming Escherichia coli Resistance Induced by the Missing SbmA Transporter System. Antimicrob. Agents Chemother..

[35] Holdsworth S.R., Law C.J. (2013). The major facilitator superfamily transporter MdtM contributes to the intrinsic resistance of Escherichia coli to quaternary ammonium compounds. J. Antimicrob. Chemother..

[36] Holdsworth S.R., Law C.J. (2012). Functional and biochemical characterisation of the Escherichia coli major facilitator superfamily multidrug transporter MdtM. Biochimie.

[37] Tan J., Huang J., Huang Y., Chen Y. (2014). Effects of single amino acid substitution on the biophysical properties and biological activities of an amphipathic α-helical antibacterial peptide against Gram-negative bacteria. Molecules.

[38] Li W., Sani M.-A., Jamasbi E., Otvos L., Hossain M.A., Wade J.D., Separovic F. (2016). Membrane interactions of proline-rich antimicrobial peptide, Chex1-Arg20, multimers. Biochim. Biophys. Acta.

[39] Mathias U., Jung M. (2007). Determination of drug-serum protein interactions via fluorescence polarization measurements. Anal. Bioanal. Chem..

