# Supplementary material for: Influence of Substitutions in the Binding Motif of Proline-Rich Antimicrobial Peptide ARV-1502 on 70S Ribosome Binding and Antimicrobial Activity
Source: Int J Mol Sci. 2022 Mar 15;23(6):3150. doi: 10.3390/ijms23063150 (PMC8950706; doi:10.3390/ijms23063150)
Supplement: Supplementary file 1 [file ijms-23-03150-s001.zip › 123 709998 combined COA.pdf]

ABclonal Science
Phone: (888) 754-5670 Fax: (888) 754-5670
Email: us.sales@abclonal.com

Quote No.	Name	Code	Peptide Sequence	Formula	M.W.	Qty	Purity	Lot NO
P18309	124	709998	Chex-RPDKPRPKFPRPRPPRPVR-NH2	C113H189N41O22	2473.90	4.1mg	88.01%	P190226-HS709998

Tests: HPLC and MS(Attached) Store at -20 degree

ABclonal warrants material of said quality at the time of sale. It is the sole responsibility of the customer to determine the adequacy of all materials for any intended or specific purpose or use. ABclonal's sole obligation is to replace the material up to the extent of the purchase price. This warranty applies only to products in original packaging and does not apply to a product which has been tampered with or altered in any way or which has been misused or damaged by accident or negligence. All claims must be received in writing (by fax or email) within 30 days from date when product arrives at the destination city. Failure to do so shall constitute a waiver by customers for all such claims.

Note : For Research Use Only

Prepared by:	Checked by:	Date
D.B(QA/QC)	P.J.(QA/QC Manager)	7/3/2019

86 Cummings Park
Woburn, MA 01801

Phone: (888) 754-5670 Fax: (888) 754-5670
Email: us.sales@abclonal.com

ABclonal Science

Phone: (888) 754-5670 Fax: (888) 754-5670
Email: us.sales@abclonal.com

MS Spectrum

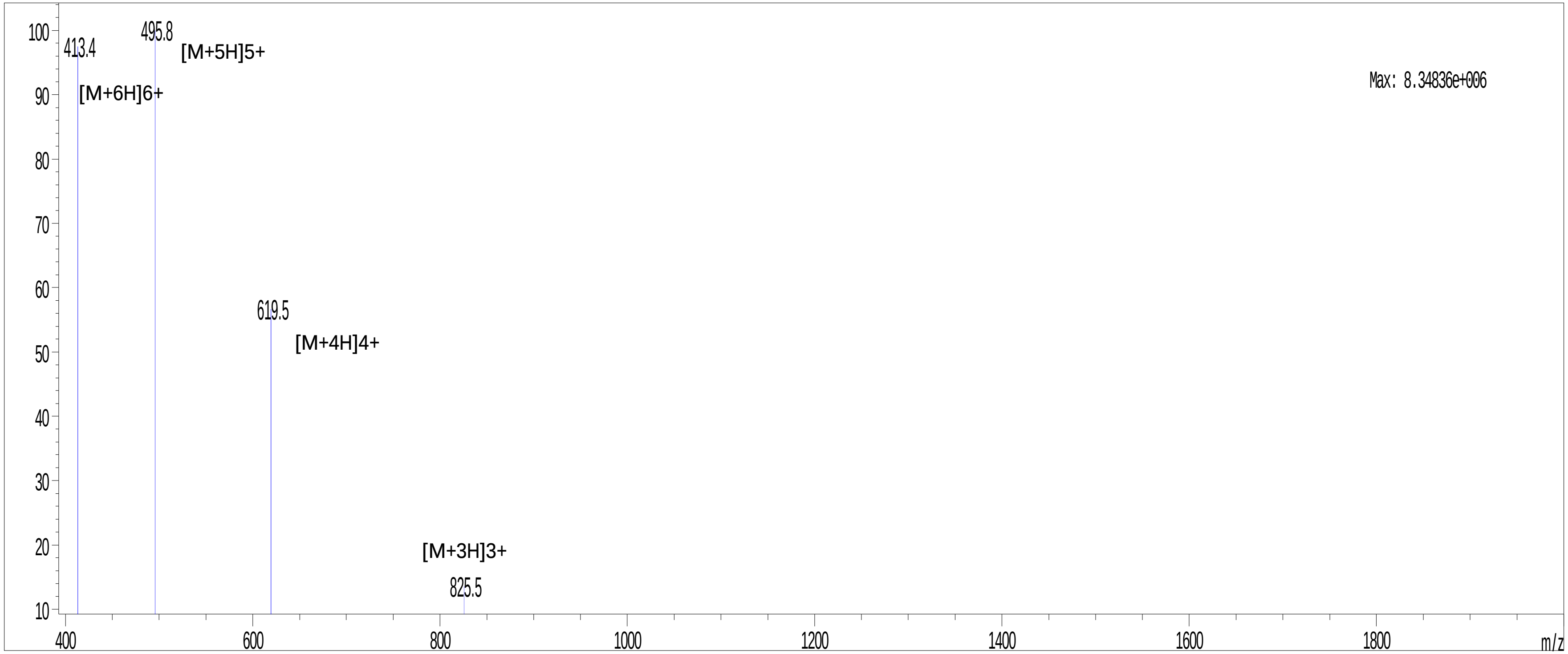

User	: YU	Probe:	ESI	Probe bias:	+4.5kv
Sample	: 124 RR-20-NH2	Nebulizer Gas Flow:	1.5L/min	Detector:	1.2kv
MW	: 2473.9	CDL:	-20.0v	T. Flow:	0.2ml/min
Lot No.	: P190226-HS709998	CDL Temp:	250℃	B. conc:	50%H2O/50%ACN
		Block Temp:	400℃		

86 Cummings Park
Woburn, MA 01801

Phone: (888) 754-5670 Fax: (888) 754-5670
Email: us.sales@abclonal.com

ABclonal Science

Phone: (888) 754-5670 Fax: (888) 754-5670
Email: us.sales@abclonal.com

Structure : 124 RR-20-NH2

Number : 010250011

Lot No : P190226-HS709998

Column : 4.6×250mm,Diamonsil 5um C18

Solvent A : 0.1% trifluoroacetic in 100% acetonitrile

Solvent B : 0.1% trifluoroacetic in 100% water

Gradient :

	A	B
0.01min	10%	90%
25min	35%	65%
25.1min	100%	0%
30min	STOP	

Flow rate : 1.0 mL/min

Wavelength : 220nm

Volume : 5ul

86 Cummings Park
Woburn, MA 01801

Phone: (888) 754-5670 Fax: (888) 754-5670
Email: us.sales@abclonal.com

ABclonal Science

Phone: (888) 754-5670 Fax: (888) 754-5670
Email: us.sales@abclonal.com

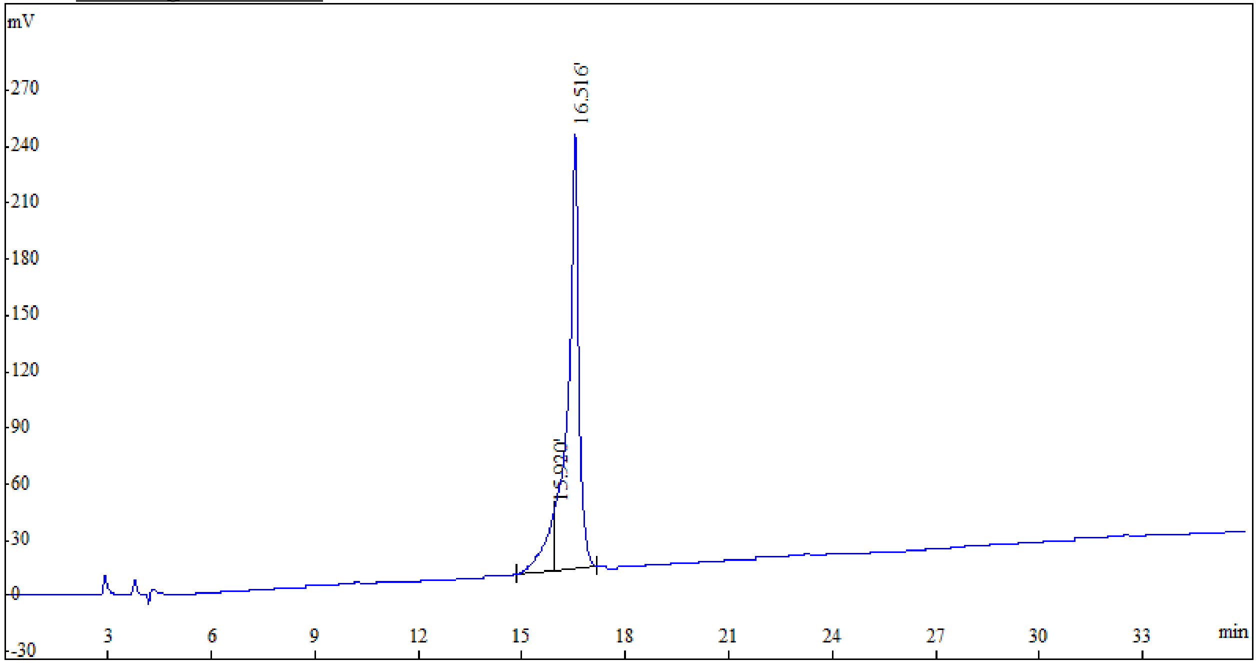

Rank	Time	Conc.	Area	Height
1	15.920	11.9863	647375	31780
2	16.516	88.0137	4753568	231042
Total		100	5400943	262822
