# Supplementary material for: Influence of Substitutions in the Binding Motif of Proline-Rich Antimicrobial Peptide ARV-1502 on 70S Ribosome Binding and Antimicrobial Activity
Source: Int J Mol Sci. 2022 Mar 15;23(6):3150. doi: 10.3390/ijms23063150 (PMC8950706; doi:10.3390/ijms23063150)
Supplement: Supplementary file 1 [file ijms-23-03150-s001.zip › 146 710026 combined COA.pdf]

ABclonal Science

Phone: (888) 754-5670 Fax: (888) 754-5670
Email: us.sales@abclonal.com

Quote No.	Name	Code	Peptide Sequence	Formula	M.W.	Qty	Purity	Lot NO
P18309	162	710026	Chex-RPDKPRPLSPRSRPPRPVR-NH2	C105H182N40O24	2388.88	4.5mg	99.17%	P190226-JQ710026

Tests: HPLC and MS(Attached) Store at -20 degree

ABclonal warrants material of said quality at the time of sale. It is the sole responsibility of the customer to determine the adequacy of all materials for any intended or specific purpose or use. ABclonal′s sole obligation is to replace the material up to the extent of the purchase price. This warranty applies only to products in original packaging and does not apply to a product which has been tampered with or altered in any way or which has been misused or damaged by accident or negligence. All claims must be received in writing (by fax or email) within 30 days from date when product arrives at the destination city. Failure to do so shall constitute a waiver by customers for all such claims.

Note : For Research Use Only

Prepared by:	Checked by:	Date
D.B(QA/QC)	P.J.(QA/QC Manager)	7/3/2019

ABclonal Science

Phone: (888) 754-5670 Fax: (888) 754-5670
Email: us.sales@abclonal.com

MS Spectrum Graph

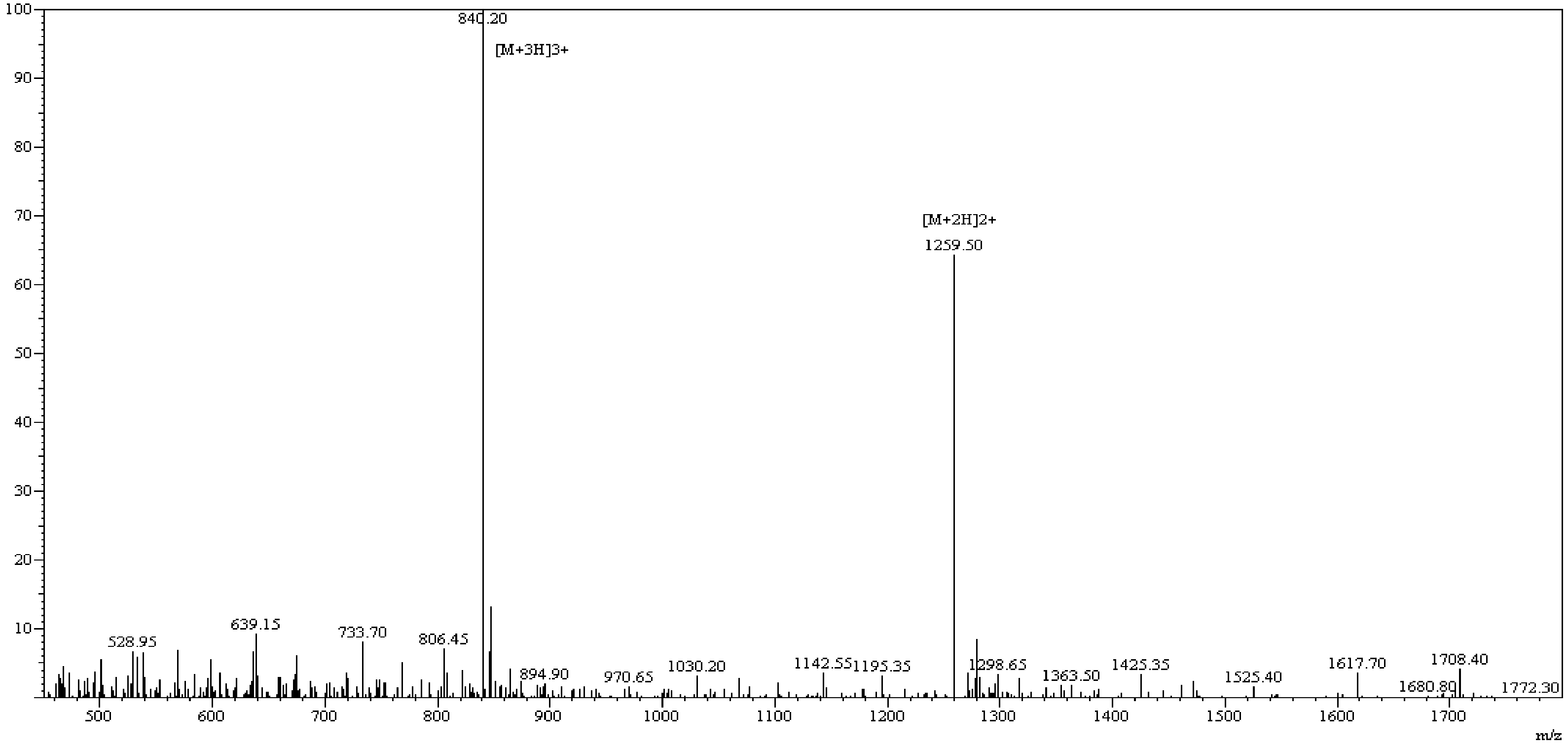

User	: SHAO	Probe:	ESI	Probe bias:	+4.5kv
Sample	: 162 RR-19	Nebulizer Gas Flow:	1.5L/min	Detector:	1.5kv
MW	: 2388.88	CDL:	-20.0v	T. Flow:	0.2ml/min
Lot No.	: P190226-JQ710026	CDL Temp:	250℃	B. conc:	50%H2O/50%ACN
		Block Temp:	200℃		

86 Cummings Park
Woburn, MA 01801

Phone: (888) 754-5670 Fax: (888) 754-5670
Email: us.sales@abclonal.com

ABclonal Science
Phone: (888) 754-5670 Fax: (888) 754-5670
Email: us.sales@abclonal.com

Structure : 162 RR-19

Number : 010250011

Lot No : P190226-JQ710026

Column : 4.6×250mm,Kromasil 100-5C18

Solvent A : 0.1% trifluoroacetic in 100% acetonitrile

Solvent B : 0.1% trifluoroacetic in 100% water

Gradient :

	A	B
0.01min	15%	85%
25min	40%	60%
25.1min	100%	0%
30min	STOP	

Flow rate : 1.0 mL/min

Wavelength : 220nm

Volume : 5ul

86 Cummings Park
Woburn, MA 01801

Phone: (888) 754-5670 Fax: (888) 754-5670
Email: us.sales@abclonal.com

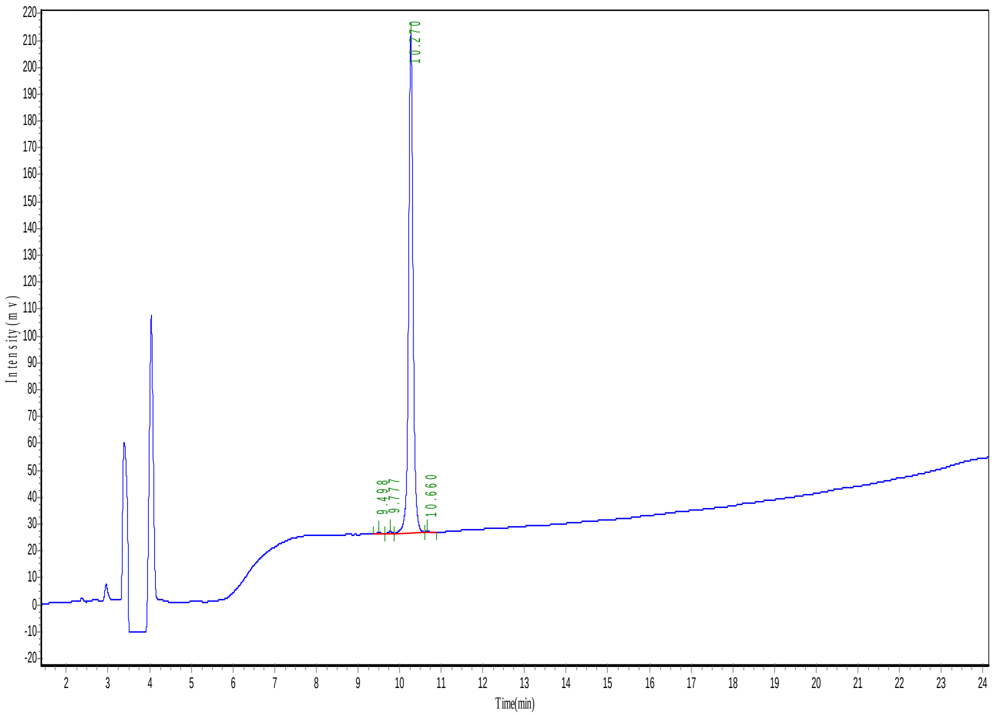

Peak No.	Ret Time	Height	Area	Conc
1	9.498	719.640	3798.562	0.2752
2	9.777	709.168	4117.707	0.2984
3	10.270	185530.172	1368732.000	99.1775
4	10.660	489.160	3434.362	0.2489
Total				100.000

86 Cummings Park
Woburn, MA 01801

Phone: (888) 754-5670 Fax: (888) 754-5670
Email: us.sales@abclonal.com
