# Supplementary material for: Influence of Substitutions in the Binding Motif of Proline-Rich Antimicrobial Peptide ARV-1502 on 70S Ribosome Binding and Antimicrobial Activity
Source: Int J Mol Sci. 2022 Mar 15;23(6):3150. doi: 10.3390/ijms23063150 (PMC8950706; doi:10.3390/ijms23063150)
Supplement: Supplementary file 1 [file ijms-23-03150-s001.zip › 174 710054 combined COA.pdf]

ABclonal Science

Phone: (888) 754-5670 Fax: (888) 754-5670
Email: us.sales@abclonal.com

Quote No.	Name	Code	Peptide Sequence	Formula	M.W.	Qty	Purity	Lot NO
P18309	190	710054	Chex-RPDKPRPYSPRDRPPRPVR-NH2	C109H180N40O26	2466.90	4.9mg	98.23%	P190226-JQ710054

Tests: HPLC and MS(Attached)   Store at -20 degree

ABclonal warrants material of said quality at the time of sale. It is the sole responsibility of the customer to determine the adequacy of all materials for any intended or specific purpose or use. ABclonal's sole obligation is to replace the material up to the extent of the purchase price. This warranty applies only to products in original packaging and does not apply to a product which has been tampered with or altered in any way or which has been misused or damaged by accident or negligence. All claims must be received in writing (by fax or email) within 30 days from date when product arrives at the destination city. Failure to do so shall constitute a waiver by customers for all such claims.

Note : For Research Use Only

Prepared by:	Checked by:	Date
D.B(QA/QC)	P.J.(QA/QC Manager)	7/3/2019

ABclonal Science

Phone: (888) 754-5670 Fax: (888) 754-5670
Email: us.sales@abclonal.com

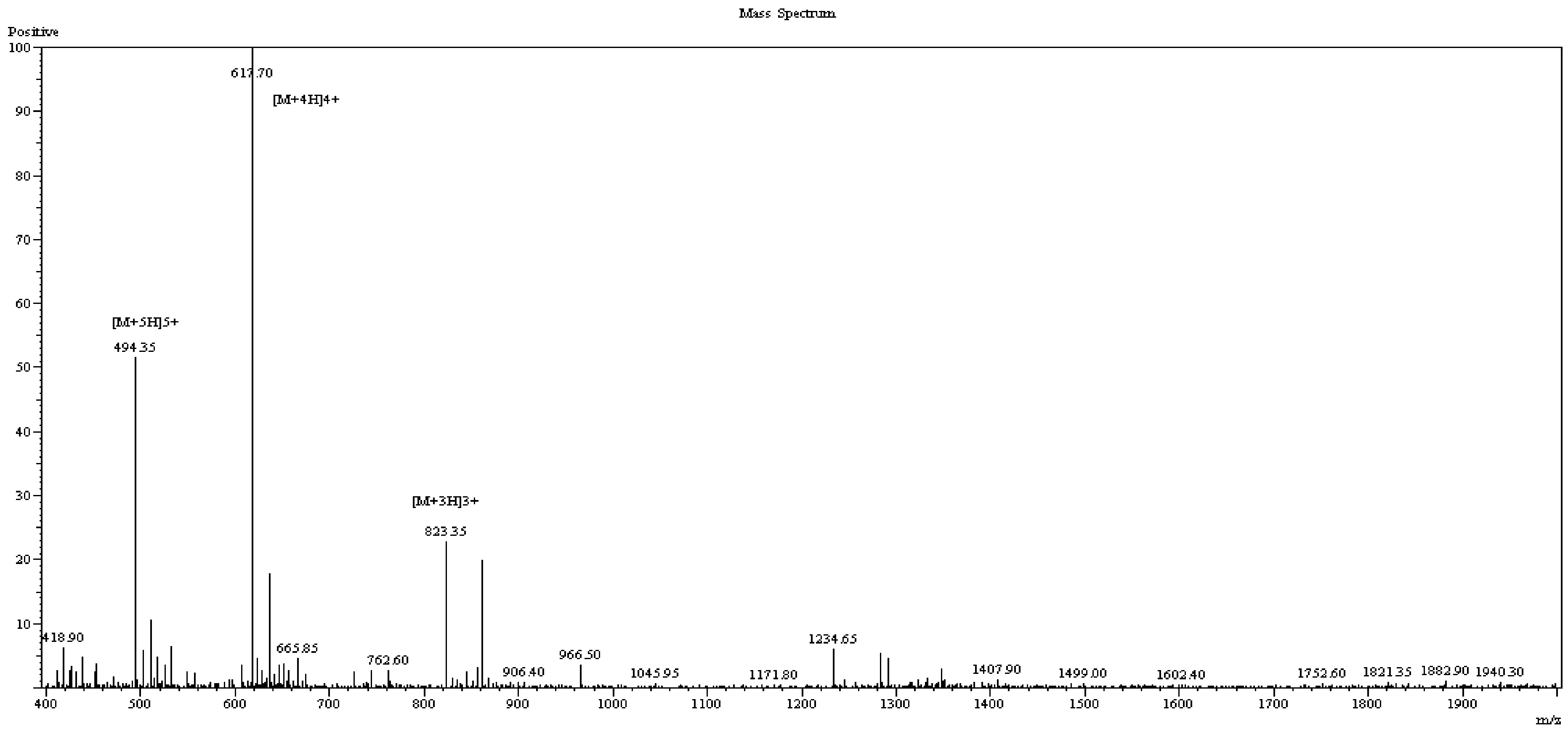

User	: shao	Probe:	ESI	Probe bias:	+4.5kv
Sample	: 190 RR-19	Nebulizer Gas Flow:	1.5L/min	Detector:	1.5kv
MW	: 2466.9	CDL:	-20.0v	T. Flow:	0.2ml/min
Lot No.	: P190226-JQ710054	CDL Temp:	250℃	B. conc:	50%H2O/50%ACN
		Block Temp:	200℃		

ABclonal Science
Phone: (888) 754-5670 Fax: (888) 754-5670
Email: us.sales@abclonal.com

Structure : 190 RR-19

Number : 010250011

Lot No : P190226-JQ710054

Column : 4.6×250mm,Kromasil 100-5C18

Solvent A : 0.1% trifluoroacetic in 100% acetonitrile

Solvent B : 0.1% trifluoroacetic in 100% water

Gradient :

	A	B
0.01min	15%	85%
25min	40%	60%
25.1min	100%	0%
30min	STOP	

Flow rate : 1.0 mL/min

Wavelength : 220nm

Volume : 5ul

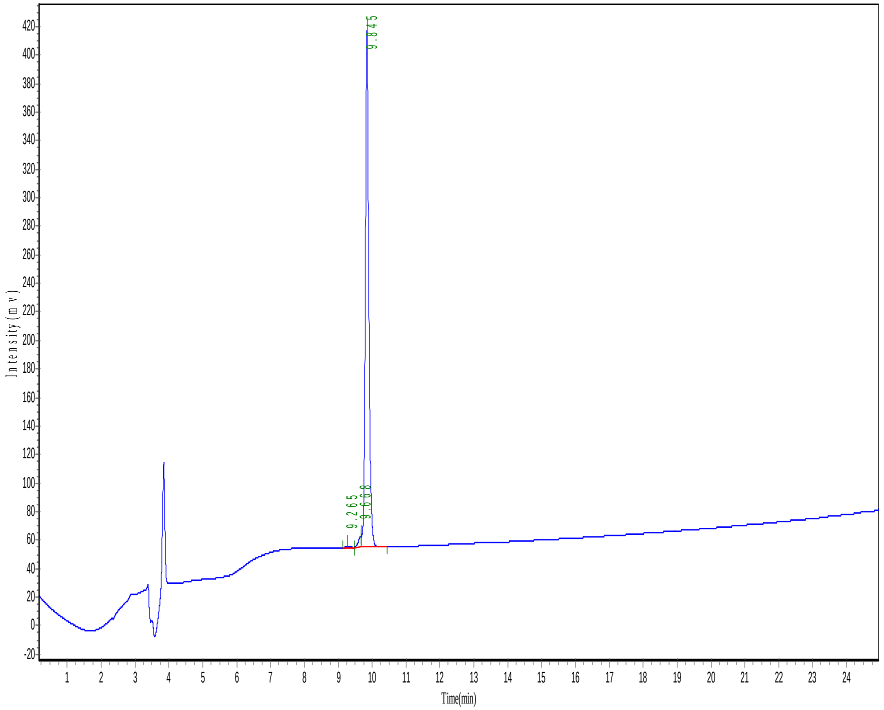

Peak No.	Ret Time	Height	Area	Conc
1	9.265	702.745	5884.186	0.2216
2	9.668	7138.807	41026.715	1.5452
3	9.845	362333.406	2608220.000	98.2332
Total				100.000
