# Supplementary material for: Influence of Substitutions in the Binding Motif of Proline-Rich Antimicrobial Peptide ARV-1502 on 70S Ribosome Binding and Antimicrobial Activity
Source: Int J Mol Sci. 2022 Mar 15;23(6):3150. doi: 10.3390/ijms23063150 (PMC8950706; doi:10.3390/ijms23063150)
Supplement: Supplementary file 1 [file ijms-23-03150-s001.zip › 182 710062 combined COA.pdf]

ABclonal Science
Phone: (888) 754-5670 Fax: (888) 754-5670
Email: us.sales@abclonal.com

Quote No.	Name	Code	Peptide Sequence	Formula	M.W.	Qty	Purity	Lot NO
P18309	198	710062	Chex-RPDKPRPYFPLPRPPRPVR-NH2	C116H185N37O23	2466.00	4.6mg	86.77%	P190226-JQ710062

Tests: HPLC and MS(Attached) Store at -20 degree

ABclonal warrants material of said quality at the time of sale. It is the sole responsibility of the customer to determine the adequacy of all materials for any intended or specific purpose or use. ABclonal's sole obligation is to replace the material up to the extent of the purchase price. This warranty applies only to products in original packaging and does not apply to a product which has been tampered with or altered in any way or which has been misused or damaged by accident or negligence. All claims must be received in writing (by fax or email) within 30 days from date when product arrives at the destination city. Failure to do so shall constitute a waiver by customers for all such claims.

Note : For Research Use Only

Prepared by:	Checked by:	Date
D.B(QA/QC)	P.J.(QA/QC Manager)	7/3/2019

86 Cummings Park
Woburn, MA 01801

Phone: (888) 754-5670 Fax: (888) 754-5670
Email: us.sales@abclonal.com

ABclonal Science
Phone: (888) 754-5670 Fax: (888) 754-5670
Email: us.sales@abclonal.com

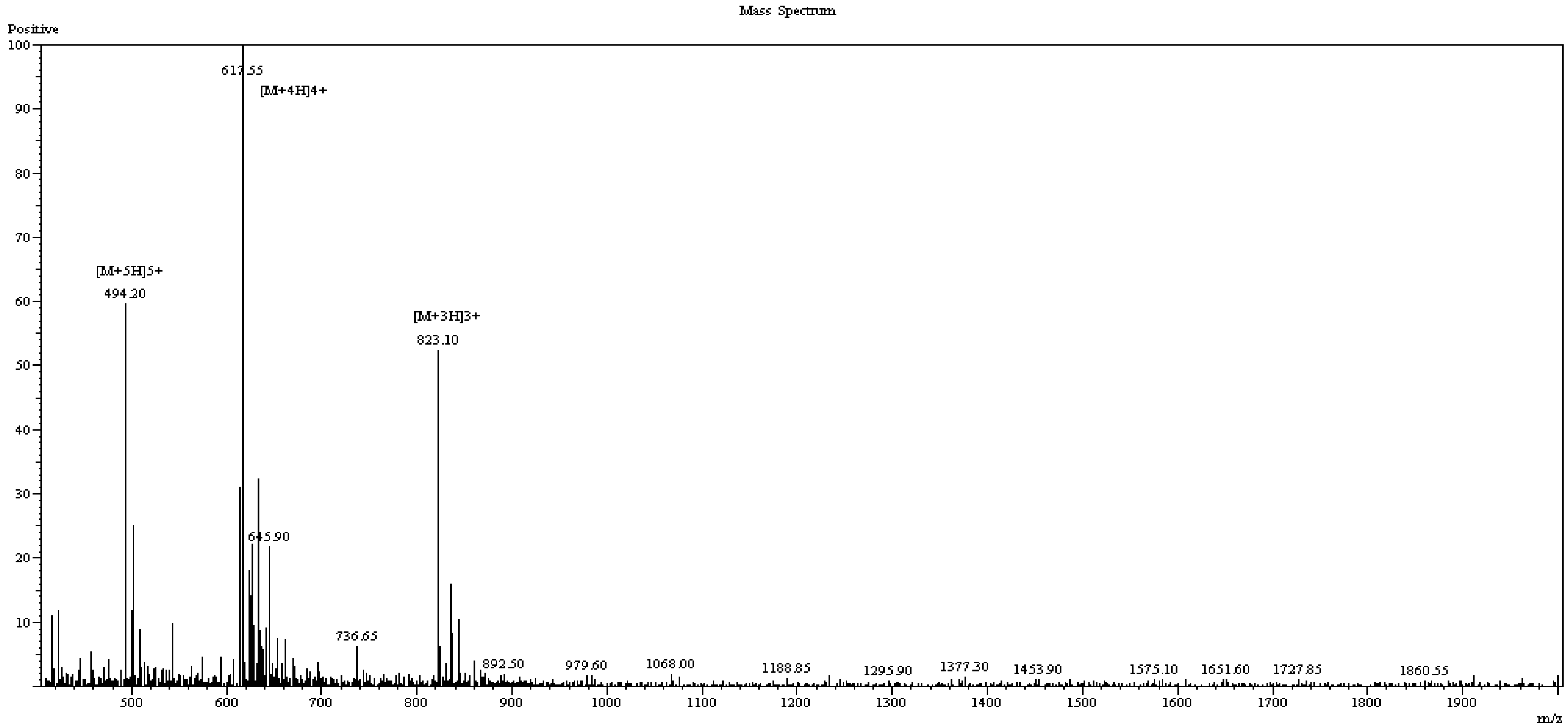

User : shao

Sample : 198 RR-19

MW : 2466

Lot No. : P190226-JQ710062

Probe: ESI

Probe bias: +4.5kv

Nebulizer Gas Flow: 1.5L/min

Detector: 1.5kv

CDL: -20.0v

T. Flow: 0.2ml/min

CDL Temp: 250℃

B. conc: 50%H2O/50%ACN

Block Temp: 200℃

86 Cummings Park
Woburn, MA 01801

Phone: (888) 754-5670 Fax: (888) 754-5670
Email: us.sales@abclonal.com

ABclonal Science
Phone: (888) 754-5670 Fax: (888) 754-5670
Email: us.sales@abclonal.com

Structure : 198 RR-19

Number : 010250011

Lot No : P190226-JQ710062

Column : 4.6×250mm,Kromasil 100-5C18

Solvent A : 0.1% trifluoroacetic in 100% acetonitrile

Solvent B : 0.1% trifluoroacetic in 100% water

Gradient :

	A	B
0.01min	15%	85%
25min	40%	60%
25.1min	100%	0%
30min	STOP	

Flow rate : 1.0 mL/min

Wavelength : 220nm

Volume : 5ul

86 Cummings Park
Woburn, MA 01801

Phone: (888) 754-5670 Fax: (888) 754-5670
Email: us.sales@abclonal.com

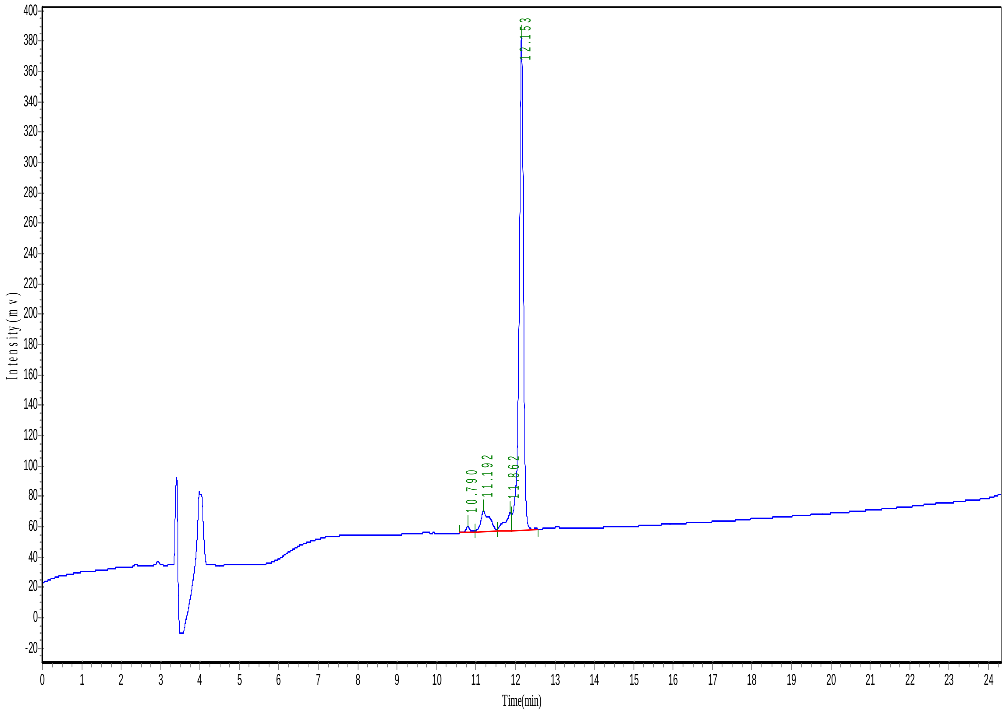

Peak No.	Ret Time	Height	Area	Conc
1	10.790	4049.940	30338.398	1.0614
2	11.192	13590.590	216818.641	7.5851
3	11.862	11349.437	131017.648	4.5835
4	12.153	324986.594	2480287.500	86.7700
Total				100.000

86 Cummings Park
Woburn, MA 01801

Phone: (888) 754-5670 Fax: (888) 754-5670
Email: us.sales@abclonal.com
