# Supplementary material for: Influence of Substitutions in the Binding Motif of Proline-Rich Antimicrobial Peptide ARV-1502 on 70S Ribosome Binding and Antimicrobial Activity
Source: Int J Mol Sci. 2022 Mar 15;23(6):3150. doi: 10.3390/ijms23063150 (PMC8950706; doi:10.3390/ijms23063150)
Supplement: Supplementary file 1 [file ijms-23-03150-s001.zip › 32 709905 combined COA .pdf]

ABclonal Science

Phone: (888) 754-5670 Fax: (888) 754-5670
Email: us.sales@abclonal.com

Quote No.	Name	Code	Peptide Sequence	Formula	M.W.	Qty	Purity	Lot NO
P18309	32	709905	Chex-RPKKPRPDLPRPRPPRPVR-NH2	C110H191N41O22	2439.94	4.1mg	94.51%	P190226-CJ709905

Tests: HPLC and MS(Attached) Store at -20 degree

ABclonal warrants material of said quality at the time of sale. It is the sole responsibility of the customer to determine the adequacy of all materials for any intended or specific purpose or use. ABclonal′s sole obligation is to replace the material up to the extent of the purchase price. This warranty applies only to products in original packaging and does not apply to a product which has been tampered with or altered in any way or which has been misused or damaged by accident or negligence. All claims must be received in writing (by fax or email) within 30 days from date when product arrives at the destination city. Failure to do so shall constitute a waiver by customers for all such claims.

Note : For Research Use Only

Prepared by: D.B(QA/QC)

Checked by: P.J.(QA/QC Manager)

Date 7/3/2019

86 Cummings Park
Woburn, MA 01801

Phone: (888) 754-5670 Fax: (888) 754-5670
Email: us.sales@abclonal.com

ABclonal Science

Phone: (888) 754-5670 Fax: (888) 754-5670
Email: us.sales@abclonal.com

MS Spectrum

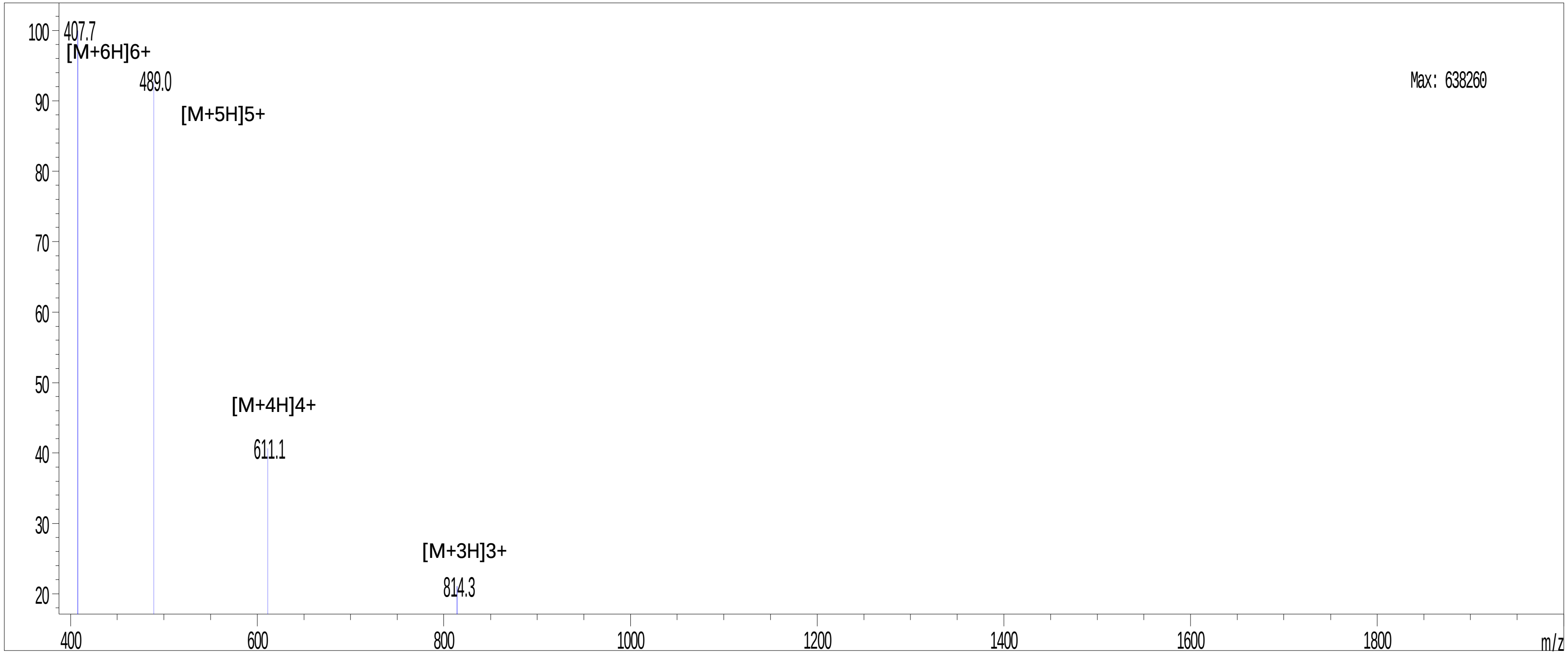

User	: YU	Probe:	ESI	Probe bias: +4.5kv
Sample	: 32 RR-19-NH2	Nebulizer Gas Flow:	1.5L/min	Detector: 1.2kv
MW	: 2439.94	CDL:	-20.0v	T. Flow: 0.2ml/min
Lot No.	: P190226-CJ709905	CDL Temp:	250℃	B. conc: 50%H2O/50%ACN
		Block Temp:	400℃	

86 Cummings Park
Woburn, MA 01801

Phone: (888) 754-5670 Fax: (888) 754-5670
Email: us.sales@abclonal.com

ABclonal Science
Phone: (888) 754-5670 Fax: (888) 754-5670
Email: us.sales@abclonal.com

Structure : 32 RR-19-NH2

Number : 010250011

Lot No : P190226-CJ709905

Column : 4.6×250mm,Diamonsil 5um C18

Solvent A : 0.1% trifluoroacetic in 100% acetonitrile

Solvent B : 0.1% trifluoroacetic in 100% water

Gradient :

	A	B
0.01min	10%	90%
25min	35%	65%
25.1min	100%	0%
30min	STOP	

Flow rate : 1.0 mL/min

Wavelength : 220nm

Volume : 5ul

86 Cummings Park
Phone: (888) 754-5670 Fax: (888) 754-5670
Woburn, MA 01801
Email: us.sales@abclonal.com

ABclonal Science
Phone: (888) 754-5670 Fax: (888) 754-5670
Email: us.sales@abclonal.com

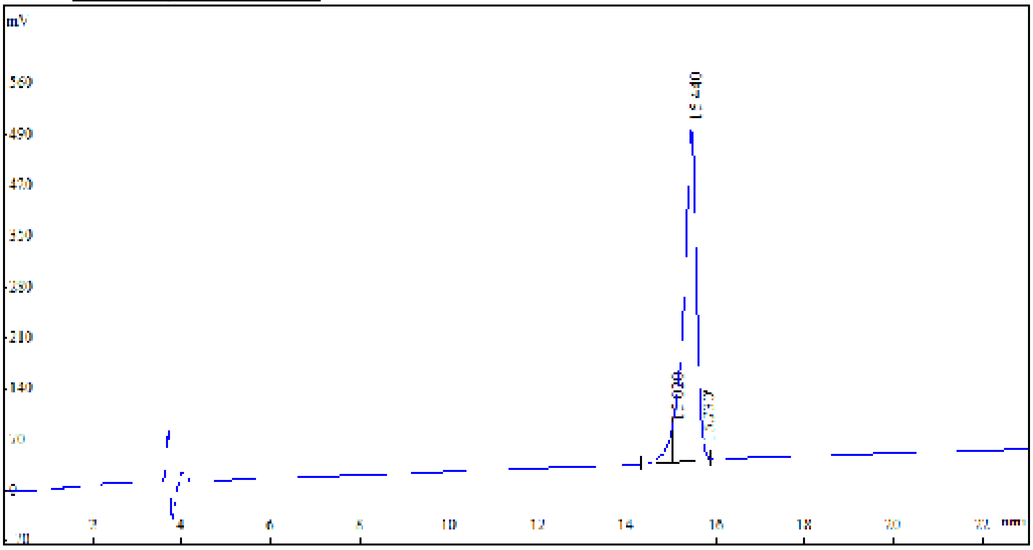

Rank	Time	Conc.	Area	Height
1	15.020	5.0251	454837	47360
2	15.440	94.5136	8554706	454760
3	15.730	0.4613	41757	14591
Total		100	9051300	516711

86 Cummings Park
(888) 754-5670 Fax: (888) 754-5670
Woburn, MA 01801
Email: us.sales@abclonal.com

Phone:
