# Supplementary material for: Influence of Substitutions in the Binding Motif of Proline-Rich Antimicrobial Peptide ARV-1502 on 70S Ribosome Binding and Antimicrobial Activity
Source: Int J Mol Sci. 2022 Mar 15;23(6):3150. doi: 10.3390/ijms23063150 (PMC8950706; doi:10.3390/ijms23063150)
Supplement: Supplementary file 1 [file ijms-23-03150-s001.zip › 42 709915 combined COA.pdf]

# ABclonal Science
Phone: (888) 754-5670 Fax: (888) 754-5670
Email: us.sales@abclonal.com

| Quote No. | Name | Code | Peptide Sequence | Formula | M.W. | Qty | Purity | Lot NO |
|---|---|---|---|---|---|---|---|---|
| P18309 | 42 | 709915 | Chex-RPKKPRPYLSRPRPPRPVR-NH2 | C113H193N41O22 | 2477.99 | 4.1mg | 89.78% | P190226-CJ709915 |
| | | | | | | | | |

Tests: HPLC and MS(Attached)    Store at -20 degree

ABclonal warrants material of said quality at the time of sale. It is the sole responsibility of the customer to determine the adequacy of all materials for any intended or specific purpose or use. ABclonal's sole obligation is to replace the material up to the extent of the purchase price. This warranty applies only to products in original packaging and does not apply to a product which has been tampered with or altered in any way or which has been misused or damaged by accident or negligence. All claims must be received in writing (by fax or email) within 30 days from date when product arrives at the destination city. Failure to do so shall constitute a waiver by customers for all such claims.

Note : For Research Use Only

Prepared by: D.B(QA/QC)    Checked by: P.J.(QA/QC Manager)    Date 7/3/2019

**ABclonal Science**
Phone: (888) 754-5670 Fax: (888) 754-5670
Email: us.sales@abclonal.com

## MS Spectrum

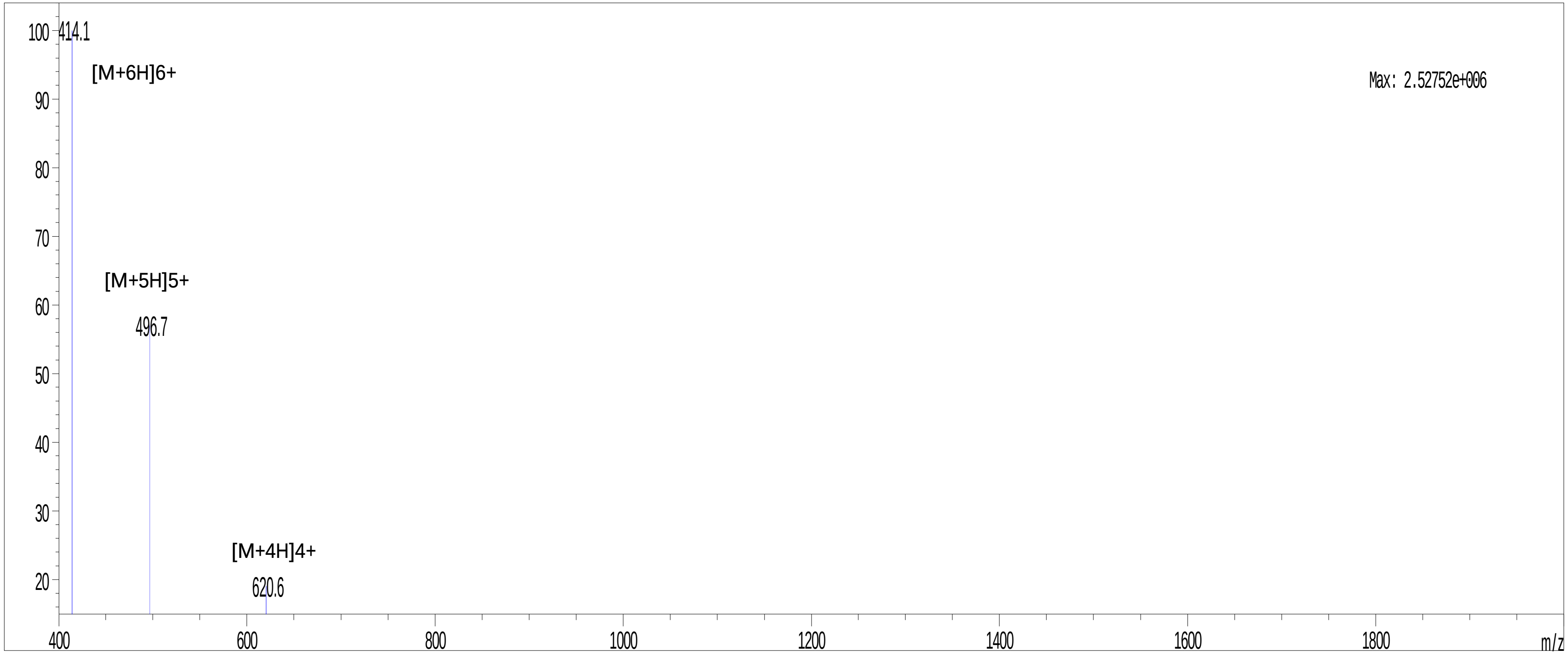


User : YU

Sample : 42 RR-19-NH2

MW : 2477.99

Lot No. : P190226-CJ709915

Probe: ESI

Probe bias: +4.5kv

Nebulizer Gas Flow: 1.5L/min

Detector: 1.2kv

CDL: -20.0v

T. Flow: 0.2ml/min

CDL Temp: 250℃

B. conc: 50%H2O/50%ACN

Block Temp: 400℃

86 Cummings Park
Woburn, MA 01801

Phone: (888) 754-5670 Fax: (888) 754-5670
Email: us.sales@abclonal.com

**ABclonal Science**
Phone: (888) 754-5670 Fax: (888) 754-5670
Email: us.sales@abclonal.com

Structure : 42 RR-19-NH2

Number : 010250011

Lot No : P190226-CJ709915

Column : 4.6×250mm,Diamonsil 5um C18

Solvent A : 0.1% trifluoroacetic in 100% acetonitrile

Solvent B : 0.1% trifluoroacetic in 100% water

Gradient :

| | A | B |
|---|---|---|
| 0.01min | 10% | 90% |
| 25min | 35% | 65% |
| 25.1min | 100% | 0% |
| 30min | STOP | |

Flow rate : 1.0 mL/min

Wavelength : 220nm

Volume : 5ul

86 Cummings Park
Phone: (888) 754-5670 Fax: (888) 754-5670
Woburn, MA 01801
Email: us.sales@abclonal.com

# ABclonal Science

Phone: (888) 754-5670 Fax: (888) 754-5670
Email: us.sales@abclonal.com

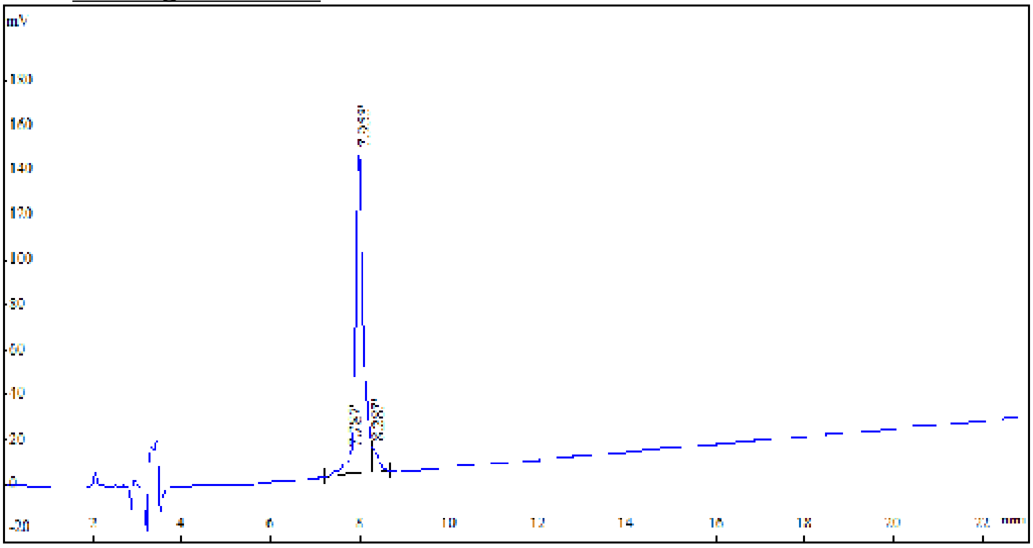


| Rank | Time | Conc. | Area | Height |
|---|---|---|---|---|
| 1 | 7.787 | 5.4686 | 97447 | 8774 |
| 2 | 7.953 | 89.7830 | 1599893 | 141990 |
| 3 | 8.287 | 4.7484 | 84615 | 10145 |
| Total | | 100 | 1781955 | 160909 |
